# Seasonal patterns of viromes in urban aquatic environments of Manitoba

**DOI:** 10.1128/aem.00408-24

**Published:** 2025-09-08

**Authors:** Jhannelle D. Francis, Kadir Yanaç, Miguel I. Uyaguari-Diaz

**Affiliations:** 1 Department of Microbiology, Faculty of Science, University of Manitoba 8664, Winnipeg, Manitoba, Canada; 2 Department of Civil Engineering, Price Faculty of Engineering, University of Manitoba 8664, Winnipeg, Manitoba, Canada; University of Delaware, Lewes, Delaware, USA

**Keywords:** wastewater, viruses, metagenomics, quantitative analyses, fecal indicator bacteria

## Abstract

Although wastewater treatment plants harbor many pathogens, traditional methods that monitor the microbial quality of surface water and wastewater have not changed since the early 1900s and often disregard the presence of other types of significant waterborne pathogens such as viruses. We used metagenomics and quantitative PCR to assess the taxonomy, functional profiling, and seasonal patterns of DNA and RNA viruses, including the virome distribution in aquatic environments receiving wastewater discharges. Environmental water samples were collected at 11 locations in Winnipeg, Manitoba, along the Red and Assiniboine rivers during the Spring, Summer, and Fall 2021. Samples were filtered and underwent skimmed milk flocculation for viral concentration. The taxonomy of classified DNA was primarily bacteriophages such as *Autographiviridae*, *Kyanoviridae*, and *Peduoviridae,* which were abundant to a lesser extent. Phage-related functionalities such as portal proteins and bacteriophage T4 major head proteins accounted for approximately 20%–40% of aquatic samples across seasons, which may possibly correspond to the DNA phages that were previously identified. RNA viruses went unclassified in the study; however, similar to DNA viruses, many were found to be non-enveloped or “naked viruses” such as *Partiviridae, Picobirnaviridae, Tombusviridae,* and *Picornaviridae,* which accounted for 3%–30% of RNA viruses in the study locations across season. Interestingly, aquatic samples were revealed to maintain an abundance of RNA phage-related functionalities such as long tail fiber protein and putative tail proteins, which accounted for approximately 2%–5% of aquatic samples during the Fall of 2021.

**IMPORTANCE:**

Municipal wastewater effluents discharged into the Red and Assiniboine rivers of Winnipeg, Manitoba, rely on traditional methods that monitor the microbial quality of effluents and receiving surface waters focus solely on the detection of coliforms, which are not necessarily good indicators of viruses or other pathogens. There is also a lack of current wastewater system effluent regulations at the federal and provincial level. Furthermore, previous literature has shown that when viral DNA and RNA sequences are blasted against current genomic databases, approximately 50% of the viral reads are classified as unknown. The significance of our research in characterizing the virome distribution in aquatic environments addresses a knowledge gap in the current effluent guidelines and a need for regulatory practices. In the long run, fecal indicator bacteria, combined with the detection of enteric viruses, may complement assessment of water quality in effluents discharged into rivers.

## INTRODUCTION

Water represents the main vehicle of dissemination chemicals and pathogens into the environment. Wastewater, defined as contaminated water from a combination of domestic, industrial, or agricultural human activities, is typically transported to wastewater treatment plants (WWTPs) for reducing organic load and pathogens. The main function of WWTPs is to enhance quality of life by safeguarding public and aquatic health. Municipalities managing WWTPs are mandated to perform routine chemical and biological tests that ensure the screening and removal of pollutants and bacteria. Microbial count is one of the most assessed wastewater parameters to determine the quality of wastewater. Treatments are designed to screen and remove a wide range of bacteria such as Proteobacteria, Bacteroidetes, Firmicutes, and Actinobacteria and waterborne parasites such as *Cryptosporidium* and *Giardia* (1).

Among Gammaproteobacteria, thermotolerant coliforms including *Escherichia coli* are the current gold standard of aquatic health when assessing the microbial quality of wastewater and receiving waters, an approach that has been used for over a century (2, 3). Canadian guidelines for effluents of WWTPs and surface waters indicate thermotolerant coliforms should not exceed 200 colony-forming units per 100 mL of sample (4). Nevertheless, its use limits the amount of information derived from a negative culture result. When a water sample is tested for the presence of fecal indicator bacteria, a negative culture result does not rule out the presence of other significant types of waterborne pathogens such as viruses or protozoans (5). Whereas a positive culture result test may be indicative of recent fecal contamination, it does not provide information on the source of contamination or on the risk to health.

Studies have suggested a rise in the number of raw wastewater discharges and pathogens present in effluent samples and aquatic environments (6–8). This could be explained due to treatment capacity at WWTPs and relying only on coliform counts to assess potential contamination. For instance, between 1998 and 2003, 18 norovirus outbreaks occurred in Finland impacting more than 10,000 residents due to inadequate wastewater treatment and further contamination of groundwaters or surface waters (9). Even though treated water samples tested negative for the presence of *E. coli* and coliforms, these outbreaks raised questions about the sole usefulness of the current microbial standards of aquatic health for routine wastewater quality testing. There are many other reports on rotavirus (Rov), crAssphage, and adenovirus prevalence in freshwaters receiving wastewater discharges (10–12). Waterborne enteric viral outbreaks occurred in groundwaters of Alberta and Ontario at 11% positive sample frequency in 2013 (13). However, some viral outbreaks go unperceived or are not reported. While there are cases of viral outbreaks, viruses in aquatic ecosystems are not studied in detail due to a lack of known viral databases. Previous literature has shown that when viral DNA and RNA sequences are blasted against viral genome databases, approximately 50% of the viral reads are classified as unknown (14, 15). Since viral DNA and RNA sequences in current genomic databases are classified as unknown, this represents an opportunity for this study to identify viral fractions for the purpose of establishing the virome distribution in aquatic environments receiving wastewater discharges. There is a need for additional water and wastewater surveillance and detection of microorganisms that this work intends to leverage. A better understanding of temporal changes of viromes (including enteric viruses) in surface waters is equally as important as current indicators of fecal contamination. Surveillance of this sentinel system is strongly suggested to be expanded to other pathogens as public health preparedness. Canada and its public health agencies should expand their surveillance capability. This study aimed to address a knowledge gap in the effluent guidelines and a need for regulatory practices beyond coliform counts. Standard methods and tools used in recent virome studies were used in our work to metagenomically characterize DNA and RNA virome distribution within the city of Winnipeg, Manitoba, and surrounding waterways. Results from the proposed study will help to be prepared for future threats to anticipate and prevent the spread of antimicrobial diseases.

## RESULTS

### High-throughput screening revealed that urban aquatic environments of Manitoba contained largely unclassified DNA viruses and, to a lesser extent, DNA bacteriophages with phage-related functionalities

**Fig 1:**
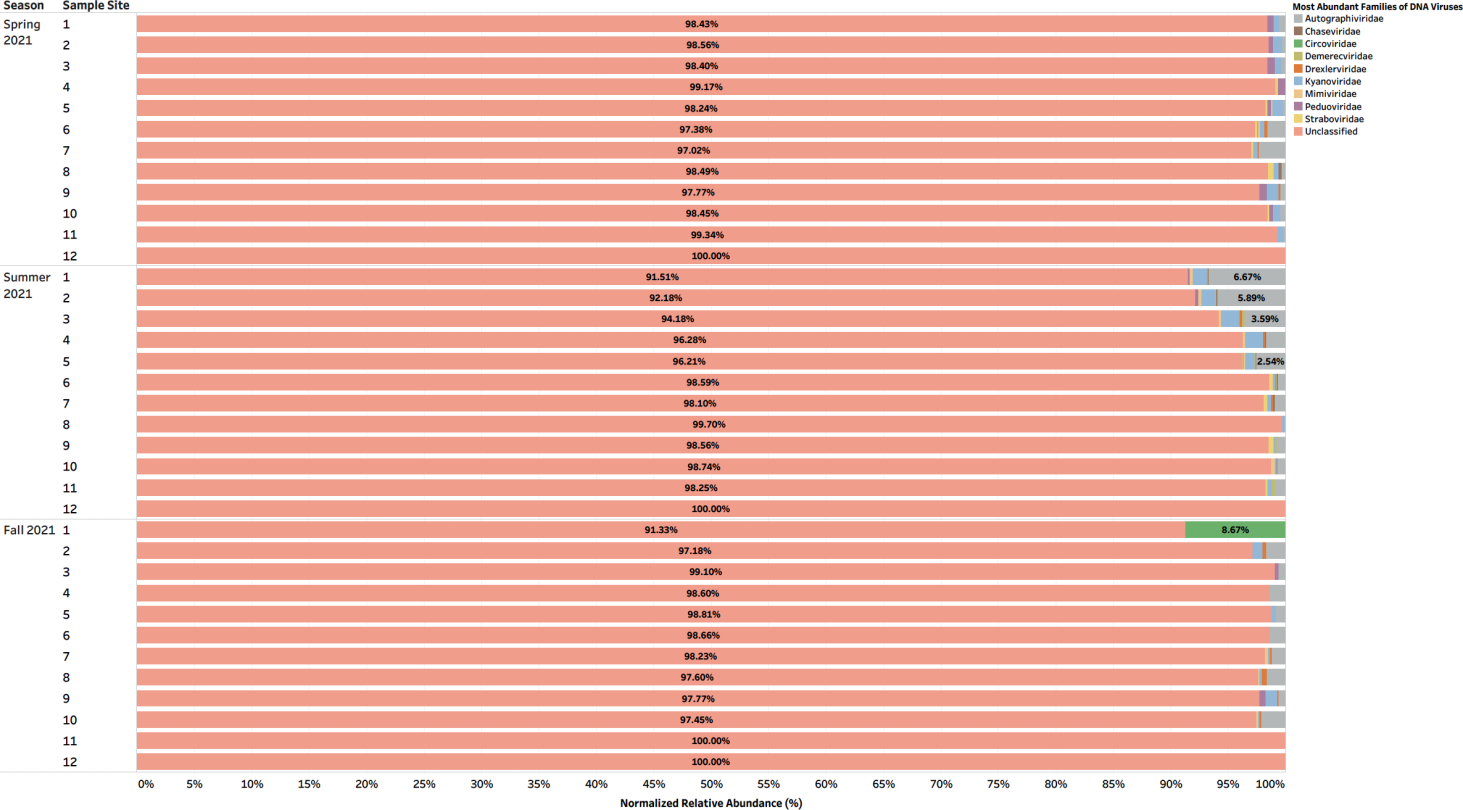
Taxonomic families of DNA viruses (inclusive of those unclassified) during the Spring, Summer, and Fall of 2021 that were identified from BLASTp v.2.15.0 against NCBI Viral RefSeq database and geNomad v.1.8.0. Host range of DNA viral families: *Autographiviridae*: bacteria (Gammaproteobacteria); *Chaseviridae*: bacteria (Gammaproteobacteria); *Circoviridae*: vertebrates and invertebrates (Cyclovirus); *Demerecviridae*: bacteria (multiple strains of *Salmonella* and *E. coli*, *Erwiniaceae*), *Drexleviridae*: plants (monocots and dicots); *Kyanoviridae*: bacteria and archaea (*Cyanobacteriota*, *Pseudomonadota*, and *Actinomycetota*); *Mimiviridae*: amoebae, protists, and algae; *Straboviridae*: gram-negative bacteria.

**Fig 2:**
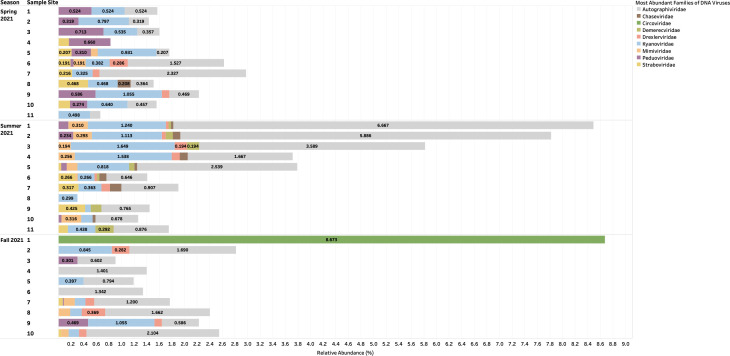
Taxonomic families of DNA viruses (without those unclassified) during the Spring, Summer, and Fall of 2021 that were identified from BLASTp v.2.15.0 against NCBI Viral RefSeq database and geNomad v.1.8.0.

**Fig 3:**
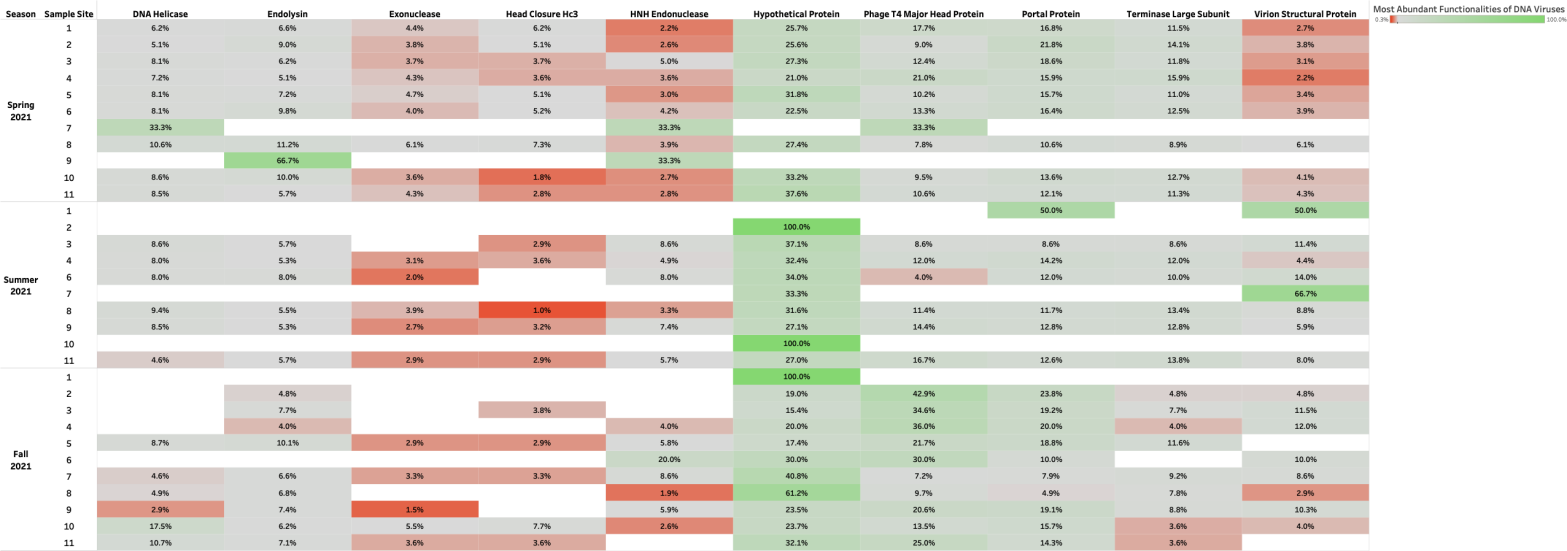
Top 10 protein-coding genes for DNA viruses during the Spring, Summer, and Fall of 2021.

Taxonomic classifications of assembled reads were confirmed as viral with geNomad v.1.8.0 and CheckV tools v.1.0.3. The viral contigs taxonomically classified using BLASTp v.2.15.0 against NCBI Viral RefSeq database (accession number: PRJNA1011997) at the family level revealed a relative abundance of unclassified DNA viruses which accounted for 90%–100% of aquatic samples collected during the Spring, Summer, and Fall of 2021 (Fig. 1). Other viruses such as *Autographiviridae*, *Kyanoviridae*, and *Peduoviridae* were abundant to a lesser extent, accounting for approximately 2%–5% % of the aquatic samples collected (Fig. 2). Assembled reads of DNA viruses were annotated with Metacerberus v.1.3 using VOG and PROG databases and revealed highest abundances (%) of the following protein coding genes: hypothetical proteins (20%–100%), bacteriophage T4 major head protein (8%–40%), and portal protein (8%–20%) in aquatic samples 1–11 collected along the Red and Assiniboine rivers (Fig. 3). Whereas these functionalities were abundant in all seasons, virion structural protein was least abundant (2%–10%), while exonuclease and head closure Hc3 were present in 80% of sample sites during Spring 2021 and in 40% of sample sites.

### RNA viruses present in urban aquatic environments of Manitoba remained stable across seasons, with a large proportion remaining unclassified and evidenced functionalities primarily related to structural pathways

**Fig 4:**
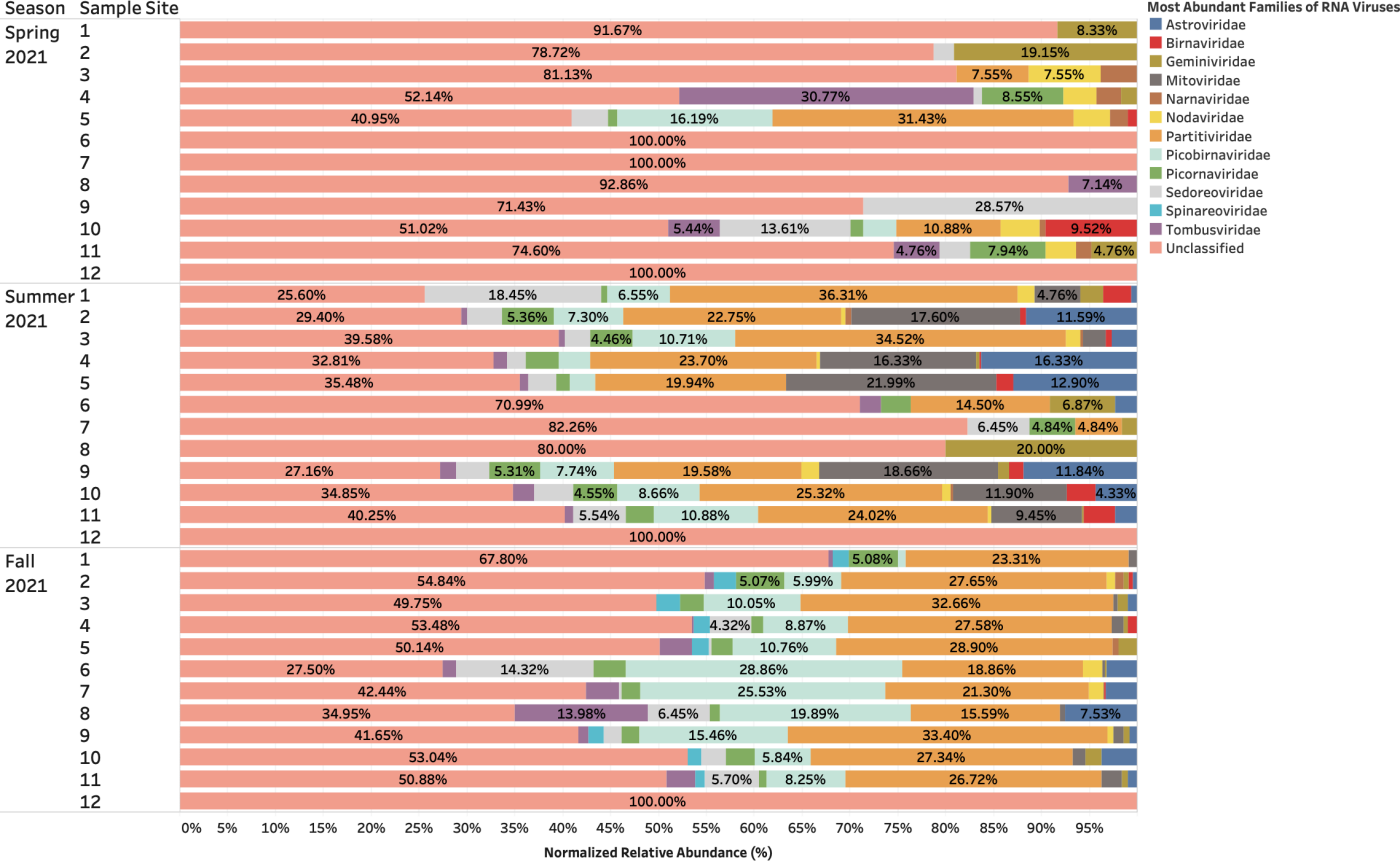
Taxonomic families of RNA viruses (without those unclassified) during the Spring, Summer, and Fall of 2021 that were identified from BLASTp v.2.15.0 against NCBI Viral RefSeq database and geNomad v.1.8.0 Host range of the RNA viral families: *Astroviridae*: humans, cattle, swine, cats, dogs, avian species; *Birnaviridae*: birds, fish, and insects; *Geminiviridae*: plants (monocots and dicots); *Mitoviridae*: plants, fungi, and invertebrates; *Narnaviridae*: fungi; *Nodaviridae*: insects (*Alphanodavirus*) or fish (*Betanodavirus*); *Partitiviridae*: plants, fungi, and protozoans; *Picobirnaviridae*: mammals, birds, reptiles, and invertebrates; *Picornaviridae*: mammals, birds, reptiles, amphibians, and fish; *Sedoreoviridae*: mammals, birds, crustaceans, arthropods, algae, and plants; *Spinareoviridae*: animals, fungi, and plants; *Tombusviridae*: plants.

**Fig 5:**
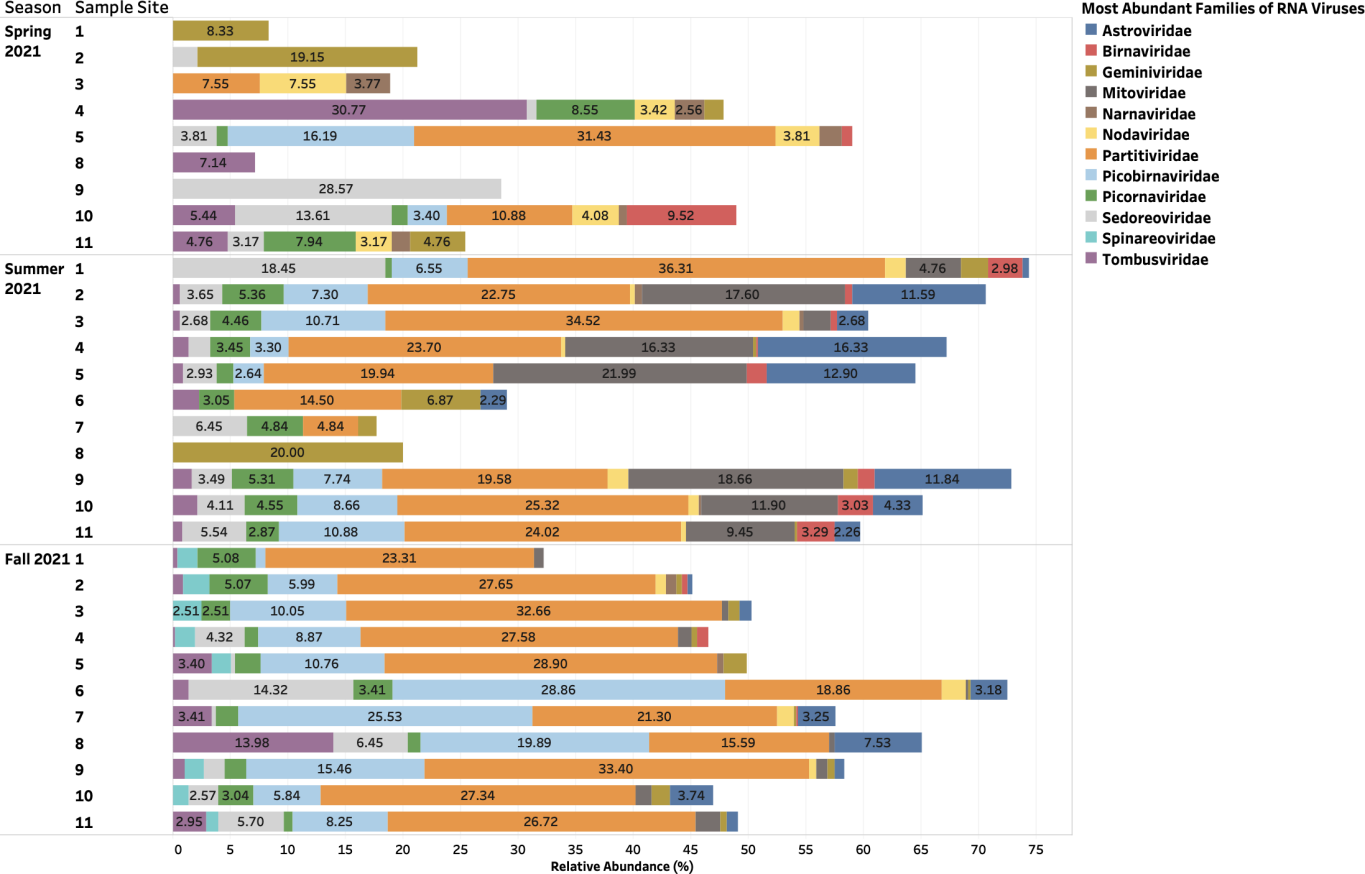
Taxonomic families of RNA viruses (without those unclassified) during the Spring, Summer, and Fall of 2021 that were identified from BLASTp v.2.15.0 against NCBI Viral RefSeq database and geNomad v.1.8.0.

**Fig 6:**
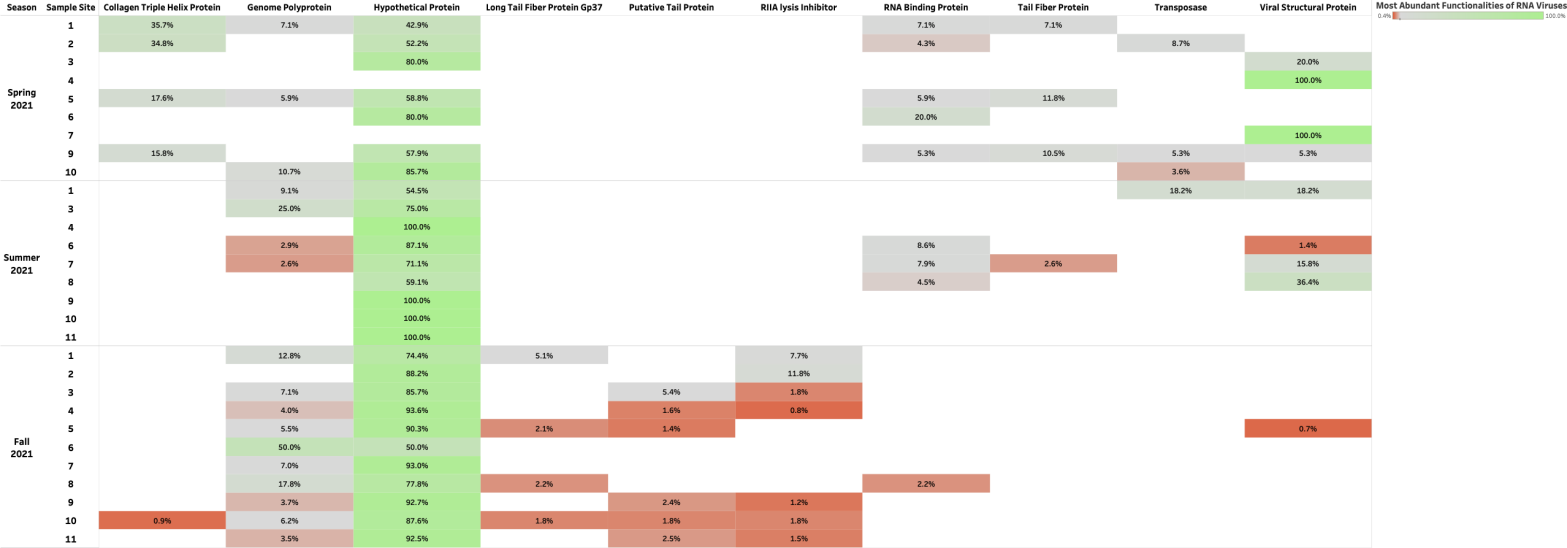
Top 10 protein-coding genes for RNA viruses during the Spring, Summer, and Fall of 2021.

A relative abundance of unclassified RNA viruses accounted for 27%–91% of samples collected during the study period (Fig. 4). Classified RNA viruses included *Partiviridae* (10%–26%), *Picobirnaviridae* (3%–10%), *Tombusviridae* (3%–30%), and *Picornaviridae* (3%–8%) (Fig. 5). Protein-coding genes identified from assembled reads of RNA viruses revealed a consistent abundance of hypothetical proteins (3%–30%) in each season from the surface water samples collected 1–11 along the Red and Assiniboine rivers (Fig. 6). Collagen triple helix protein (15%–35%) and viral structural protein (18%–100%) were found to be most abundant during the Spring of 2021, while long tail fiber protein (1%–5%), putative tail protein (2%–5%), and RIIA lysis inhibiter (1%–11%) were only found to be present during the Fall of 2021 (Fig. 6). Our findings suggest that the protein-coding genes for DNA viruses appeared to be more stable throughout each sample site across seasons, while those for RNA viruses were found to be seasonally variable.

Unclassified viromes were present in all water samples with a higher percentage of viral DNA remaining unclassified compared to viral RNA. Without the presence of unclassified viruses, we observed that both the DNA and RNA viruses remained stable and consistent throughout changes across seasons. A higher abundance of RNA viruses was observed during the Summer and Fall of 2021, while that of DNA viruses was observed during the Fall and Summer of 2021.

### Exploratory factor analysis (EFA) (orthomax rotation) revealed that day length and temperature were significant factors affecting relative abundances of urban viral communities in Manitoba

**Fig 7:**
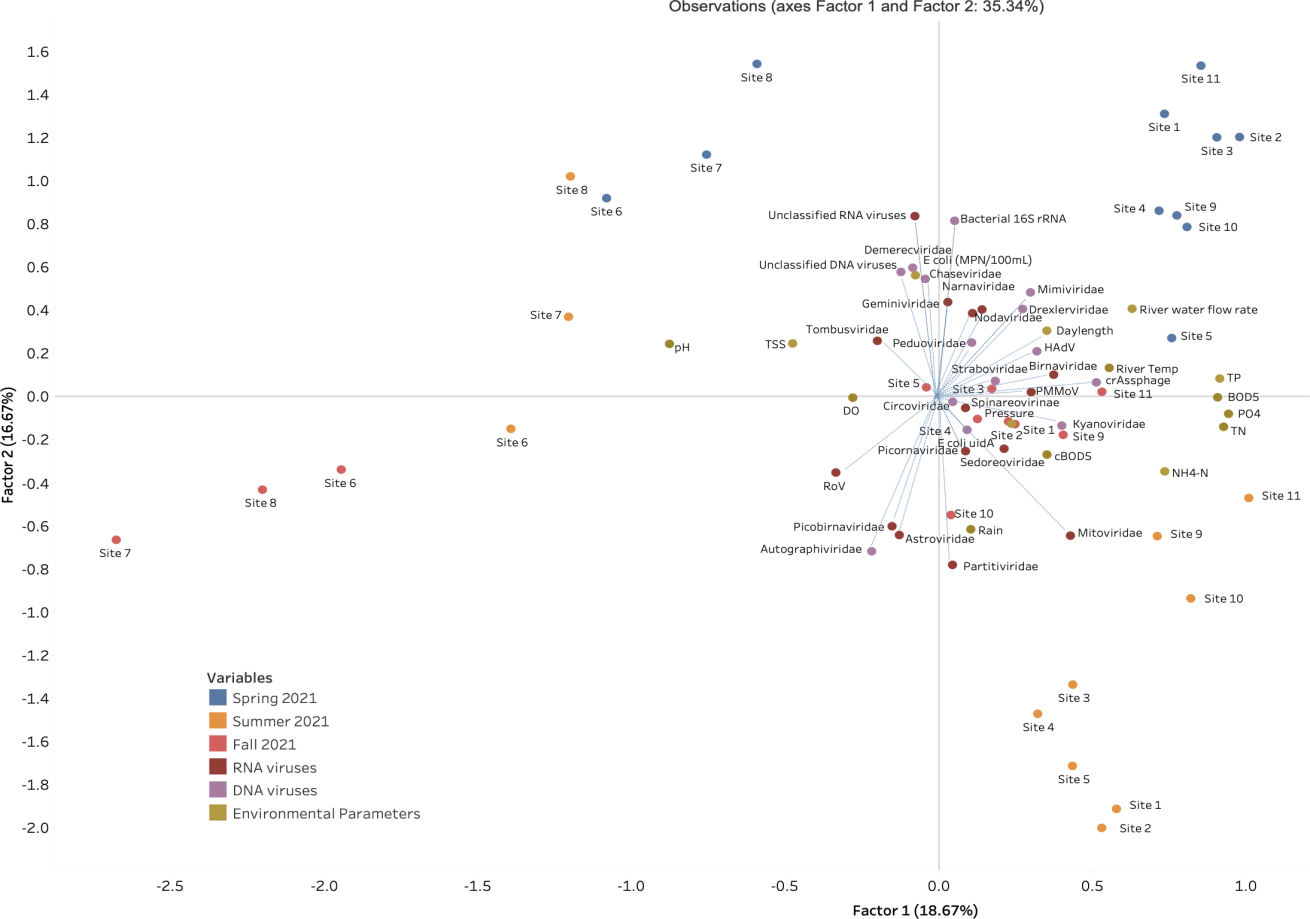
Factor analysis of viral DNA and RNA families and environmental variables observed at each sample collection site (1–11) during the Spring (blue dots), Summer (orange dots), and Fall (red dots) of 2021. Factor 1 represents the influence of agricultural activities from rural environments, while factor 2 represents the effect of urbanization and industrialization practices on aquatic environments.

Factor 1 or “land use” and factor 2 or “day length and temperature” accounted for 18.67% and 16.67% of the observed variability, respectively. Approximately 50% of the data were explained by three factors combined (Fig. 7; Fig. S2 and S3). Three distinct clusters of sites 6, 7, and 8 from the Assiniboine River are each visually identified for the Spring, Summer, and Fall of 2021 sampling events (Fig. 7). The remaining sample locations 1–5 and 9–11 from the Red River formed individual clusters for the Spring and Summer 2021 collection events, while those collected during the fall were scattered (Fig. 7). Factor loadings for the environmental parameters—river water flow rate, day length, and the temperature of river—all fell within the same cluster as sites 1–5 and 9–11 for the Spring 2021 sample collection. On the other hand, rain, pressure, total nitrogen (TN), PO_4_, NH_4_-N, cBOD_5_, and BOD_5_ were within the same quadrant as sites 1–5 and 9–11 collected during Summer 2021 (Fig. 7).

**Fig 8:**
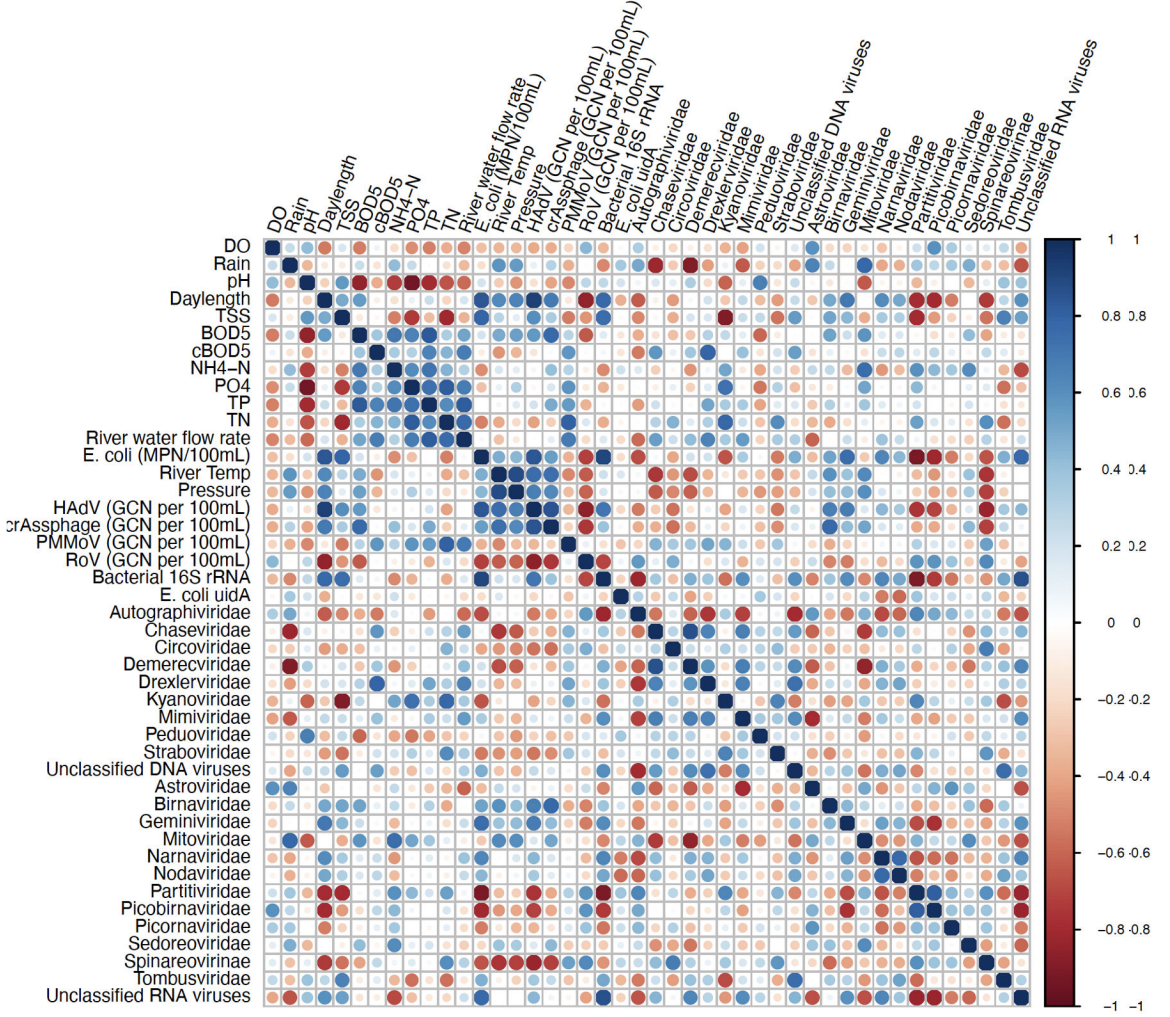
Correlogram of viral DNA and RNA families and environmental variables observed at each sample collection site (1–11). Correlation coefficients range from +1 to −1 and are represented by circles colored blue to red. DO, dissolved oxygen; River TEM, temperature of the river water; cBOD_5_, carbonaceous biochemical oxygen demand; BOD_5_, biochemical oxygen demand; NH_4_-N, ammonia, PO_4_, ortho-phosphorus; *E. coli* (MPN/100 mL), *E. coli* colony forming unit counts. The following viruses were assessed through quantitative analyses (GCN per 100 mL), HAdV, crAssphage, PMMoV, and RoV, while the remaining viruses were assessed metagenomically.

Human adenovirus (HAdV) was significantly impacted by most of the environmental parameters. A positive correlation was observed for the following water quality parameters: day length (*r* = 0.80689, *P* = 1.00E − 04), BOD_5_ (*r* = 0.52108, *P* = 1.9E − 03), bacterial counts (*r* = 0.62409, *P* = 1.00E − 04), and temperature of river (*r* = 0.57531, *P* = 5.00E − 04) (Fig. 8). crAssphage was also found to be significantly impacted and positively correlated to cBOD_5_ (*r* = 0.62909, *P* < 1.00E − 04) and total phosphorus (TP) (*r* = 0.50930, *P* = 2.5E − 03). Other DNA viruses were found to be negatively correlated and significantly impacted by the following environmental parameters: *Autographiviridae* was most impacted by river water flow rate (*r* = −0.51172, *P* = 2.3E − 03), *Chaseviridae* was impacted by rain (*r* = 0.50531, *P* = 2.7E − 03) and the temperature of the river (*r* = −0.50984, *P* = 2.4E − 03), while *Kyanoviridae* was most impacted by total suspended solids (TSS) (*r* = −0.69565, *P* < 1.00E − 04) (Fig. 8).

Similar to DNA viruses, the abundance of many classified RNA viruses was influenced by differing parameters. The presence of pepper mild mottle virus (PMMoV) in surface waters was found to be significantly impacted and positively correlated to TN (*r* = 0.51643, *P* = 2.1E − 03), while the abundance of RoV was evidenced to be negatively influenced by day length (*r* = −0.63542, *P* < 1.00E − 04) (Fig. 8). The abundance of *Picobirnaviridae* and *Partitiviridae* was found to be strongly influenced by day length and *E. coli* counts (MPN/100 mL) (*r* = −0.58117, *P* = 4.00E − 04; *r* = 0.56024, *P* = 7.00E − 04 and *r* = −0.65360, *P* < 1.00E-04; *r* = −0.64822, *P* < 1.00E − 04), respectively. *Partitiviridae* was also negatively impacted by TSS (*r* = −0.55776, *P* = 7.00E − 04). Day length was also found to have a negative influence on *Spinareoviridae* (*r* = −0.51313, *P* = 2.3E − 03), while *E. coli* counts (MPN/100 mL) were also found to be positively correlated with unclassified RNA viruses (*r* = 0.64555, *P* < 1.00E − 04). NH_4_-N was observed to have a significant and positive impact on *Mitoviridae* (*r* = 0.59617, *P* = 3.00E-04), while negatively correlating to unclassified RNA viruses NH_4_-N (*r* = −0.51840, *P* = 2.0E − 03) (Fig. 8). Whereas the abundance of many DNA and RNA viruses was strongly influenced by water quality parameters, the majority of classified RNA viruses (53.3%) were not strongly correlated to any of the water quality parameters assessed in comparison to 41.6% of DNA viruses (Fig. 8). Interestingly, pH and DO did not seem to have a major influence on the abundance of viruses in the aquatic bodies studied here. Rain had the least impact on the viral community, while day length and temperature significantly affected most DNA and RNA viruses in this study. Expectedly, bacterial *16S rRNA* was significantly positively correlated to *E. coli* counts (MPN/100 mL) (*r* = 0.697, *P* < 1.00E − 04).

### Observational trends during the fall suggest that gene copies for each marker were consistent across all sample locations, while those quantified for the spring and summer collection events fluctuated across sample sites

**Fig 9:**
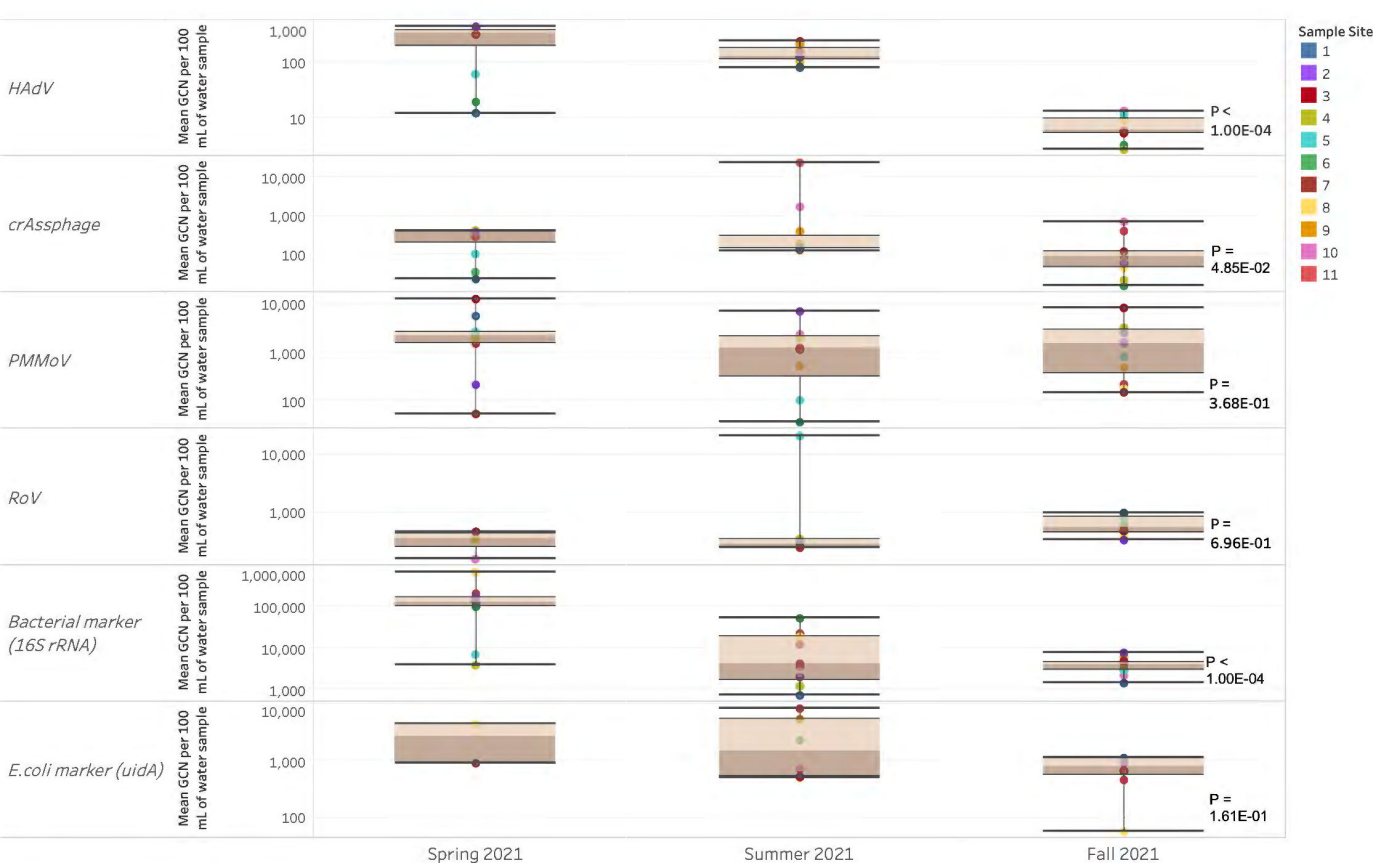
Gene copy numbers per volume (100 mL) of viral (HAdV, crAssphage, PMMoV, and RoV) and bacterial (*uidA* and *16S rRNA*) markers during the Spring, Summer, and Fall of 2021 at 11 locations along the Red and Assiniboine rivers, Winnipeg, MB. Number on the lower right represents *P*-value from the PROC mixed with repeated measures. Statistical significance was set at the 0.05 level.

Longitudinal analysis with repeated values was conducted on the qPCR data for the DNA and RNA viral markers of interest, HAdV, crAssphage, PMMoV, and RoV, as well as the bacterial marker (*16S rRNA*) and *E. coli* marker (*uidA*), to detect differences across seasons. With the exception of PMMoV, RoV, and *E. coli,* gene copies of the viral and bacterial markers quantitatively assessed at each sample collection site were all found to change across season (*P*-value = 1.00E − 04) (Fig. 9). Although consistent quantities per volume of RoV and crAssphage were observed in orders of <10^3^, location 5 contained strikingly high amounts of the virus in the order of 10^4^, which was quantified during the Summer (Fig. 9; Fig. S11). Bacterial gene copy numbers (GCNs) of *16S rRNA* and *uidA* were observed over time in orders of magnitude ranging from 10^6^ and 10^4^ (volume), respectively. GCNs per volume were lower for the DNA enteric virus HAdV in orders of 10^2^. When compared to Spring 2021, a significant reduction in gene copies (*P*-value = 1.00E − 04) was observed during Summer and Fall of 2021.

### Beta diversity of DNA and RNA virome distribution was influenced by seasonality

**Fig 10:**
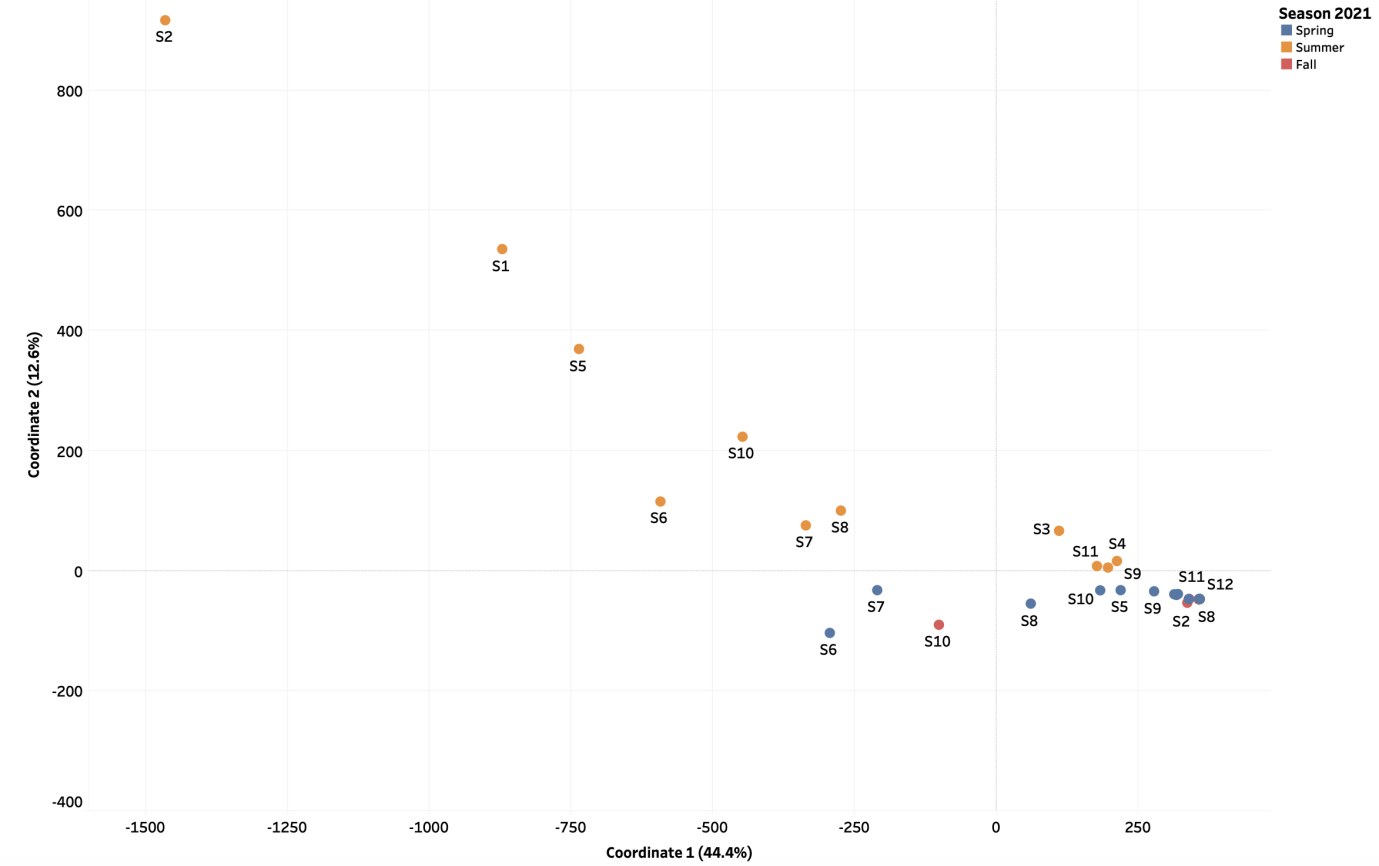
Assessment of the beta diversity among DNA viral communities present in aquatic samples 1–11 collected during the Spring (blue dots), Summer (orange dots), and Fall (red dots) 2021. The Bray-Curtis dissimilarity (beta diversity) index was calculated using the k-mer tables generated by MerCat2 with factoextra and MASS packages in RStudio.

**Fig 11:**
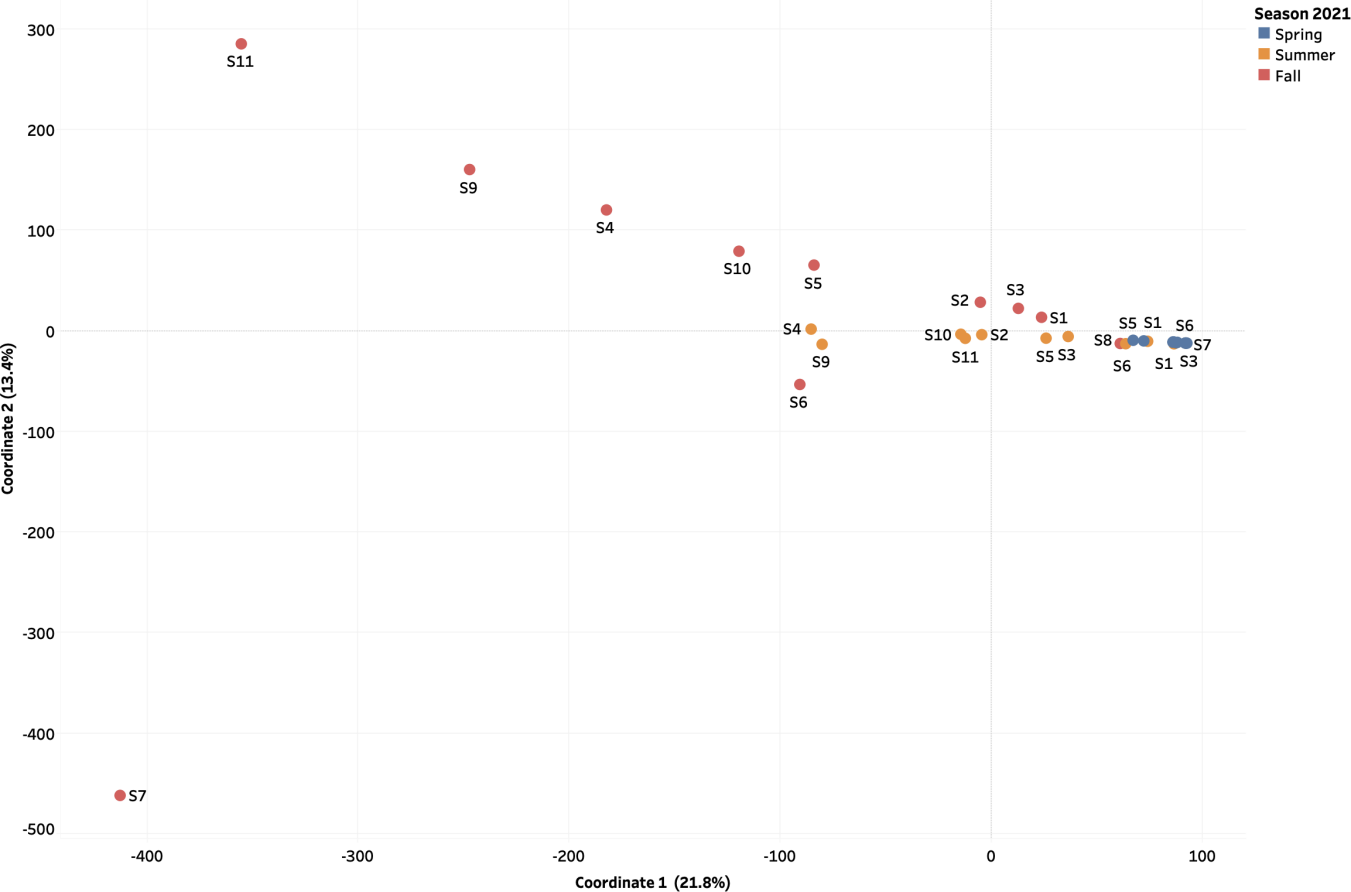
Assessment of the beta diversity among RNA viral communities present in aquatic samples 1–11 collected during the Spring (blue dots), Summer (orange dots), and Fall (red dots) 2021. The Bray-Curtis dissimilarity (beta diversity) index was calculated using the k-mer tables generated by MerCat2 with factoextra and MASS packages in RStudio.

In 2021, the populations of the DNA viruses present during the Spring and Fall were evidenced to be more closely related (low Bray-Curtis dissimilarity distance of 5.73E − 02) compared to those present in the Summer (Fig. 10). Observational trends suggest that 10 out of the 11 sample sites in the study contained viral DNA community during the Fall of 2021 that were closely related to all (100%, 11 out of 11) of the sample sites that contained viral DNA identified during the Spring. DNA viruses observed in Summer appeared to be more diverse from those identified during the Spring and Fall, with a high Bray-Curtis dissimilarity distance of −11.90 and −10.44, respectively (Fig. 10). In contrast, RNA viral communities identified during Spring appeared to be most similar and closely related (a low Bray-Curtis dissimilarity distance of 2.10) to those identified during the Summer of 2021 (Fig. 11). RNA viruses identified during the Fall of 2021 were the most diverse from those identified in the Summer and Spring of 2021 (high Bray-Curtis dissimilarity distance of 12.90 and 2.21, respectively).

### Alpha diversities Simpson’s (1-D) and Shannon (H) reveal seasonal patterns among DNA and RNA viral communities in urban Manitoba

**Fig 12:**
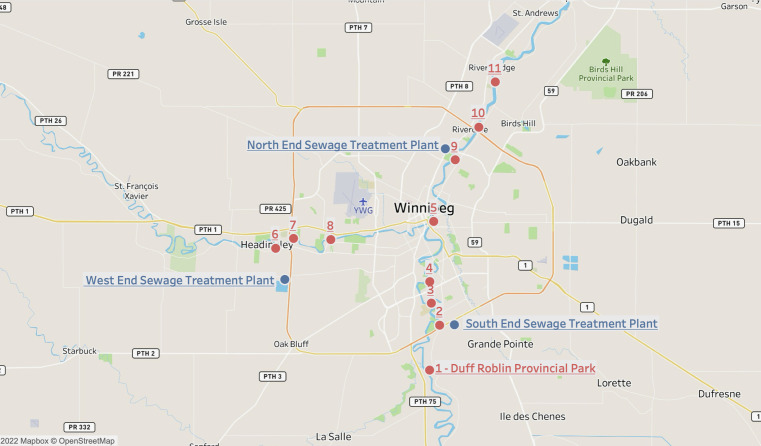
Map of 11 environmental sample locations indicated by the red dots. Winnipeg’s three major sewage treatment plants are indicated in blue as one of the sites visited even though no sampling events occurred. Source: Google Maps and Tableau Desktop (version 2024.2).

Using the Simpson’s (1-D) diversity index, DNA viral communities present in approximately 54.54% (6/11) surface water samples collected along the Red River evidenced more species diversity (with site 8 representing the highest diversity) during the Fall of 2021. Viral DNA communities present in the south end (locations numbered 1 and 2) and in the north end (location numbered 11) of the Red River as well as in the Assiniboine River (location 8) during Summer of 2021 were less diverse (Tables 1 and 2; Fig. 12; Fig. S4). Not only were species diversity found to be highest (0.9800) in the Red River at site 9 during the fall, but also lowest (0.5000) in the Assiniboine River at location 6. To further confirm the species diversity and richness observed in the sequenced samples, the Shannon diversity index was measured. In contrast to Simpson’s (1-D) diversity index, the DNA populations present in each surface water sample except for location 7 in the Assiniboine River collected during the Summer of 2021 measured high values of Shannon diversity index, which indicated high species diversity present in these samples (Tables 1 and 2; Fig. 12; Fig. S5). However, during Fall 2021, DNA viral communities showed lower values of Shannon diversity index. Therefore, the alpha diversity values suggest that viral DNA populations have seasonal patterns with a higher species evenness during the summer and a higher richness component during the fall. Although the results of a PROC GLM test for Shannon and Simpson diversity indices revealed that there were no statistical differences (*P*-value ≥1.661E – 01) in the diversity of species of DNA viruses between sample locations, statistical differences (*P*-value ≤ 1.00E − 04) in species diversities of DNA viruses were observed in all seasons. Similar to the viral DNA community, results of a PROC GLM test for Shannon and Simpson diversity indices for the RNA viral community indicated statistical differences (*P*-value = 1.00E − 04) in the abundance and richness of RNA viruses collected across seasons with no major differences (*P*-value ≥1.026E − 01) observed between locations. Both DNA and RNA viral communities exhibited fluctuations between locations, with RNA populations depicting a greater degree of variability (Table 1; Fig. S2 through S4).

**TABLE 1:** Assessment of alpha diversity indices: Simpson (1-D) and Shannon (H) among DNA and RNA viral communities present in samples 1–11 collected along the Red and Assiniboine rivers of Winnipeg during the Spring, Summer, and Fall of 2021^*a*^

Location	Simpson (1-D) vDNA			Simpson (1-D) vRNA			Shannon (H) vDNA			Shannon (H) vRNA		
	SP	SUM	FALL	SP	SUM	FALL	SP	SUM	FALL	SP	SUM	FALL
1	0.96	0.79	0.93	0.99	0.96	0.93	10.03	18.42	13.09	10.09	11.46	13.07
2	0.91	0.74	0.83	0.85	0.9	0.9	14.76	18.66	13.45	10.1	13.64	13.38
3	0.9	0.88	0.94	0.95	0.9	0.9	14.8	16.85	10.03	9.56	12.88	13.06
4	0.89	0.9	0.96	0.98	0.88	0.86	14.61	16.26	9.68	9.72	14.31	14.53
5	0.89	0.79	0.96	0.88	0.93	0.87	16.21	18.32	8.53	11.58	13.24	14.06
6	0.86	0.82	0.5	0.93	0.91	0.87	17.91	18.24	8.1	7.68	12.04	14.03
7	0.83	0.83	0.72	0.99	0.87	0.87	17.7	17.95	18.8	6.53	9.91	14.93
8	0.86	0.83	0.9	0.98	0.9	0.94	17	17.82	10.54	7.48	7.44	12
9	0.88	0.9	0.98	0.98	0.92	0.87	15.55	16.41	8.95	7.47	14.33	14.72
10	0.88	0.81	0.79	0.96	0.93	0.9	16.49	18.05	17.36	11.6	13.81	14.33
11	0.95	0.89	0.98	0.92	0.94	0.89	13.86	16.53	9.04	9.94	13.78	14.96

^*a*^ SP, spring; SUM, summer; FALL, fall; vDNA: DNA viruses; vRNA: RNA viruses.

**TABLE 2:** Coordinates for the 11 sample locations and Winnipeg’s three major sewage treatment plants

Sample collection location	Latitude coordinate	Longitude coordinate	Description
1 - Duff Roblin Provincial Park	49.7539296	−97.1314251	Upstream to all WWTPs
2 - Red River (South-end)	49.7942764	−97.1174512	Downstream to SEWPCC
3 - Red River (South-end)	49.8141103	−97.1289894	Downstream to SEWPCC
4 - Red River (South-end)	49.8334281	−97.1311847	Downstream to SEWPCC
5 - Red River (Central)	49.8874423	−97.1257154	Downstream to SEWPCC
6 - Assiniboine River	49.8632965	−97.3456944	Upstream to WEWPCC
7 - Assiniboine River	49.8721853	−97.3208206	Downstream to WEWPCC
8 - Assiniboine River	49.8711465	−97.2689454	Downstream to WEWPCC
9 - Red River (North-end)	49.9427026	−97.0957565	Upstream to NEWPCC
10 - Red River (North-end)	49.971833	−97.0628907	Downstream to NEWPCC
11 - Red River (North-end)	50.0123769	−97.0399759	Downstream to NEWPCC
North End Sewage Treatment Plant	49.95801726	−97.10907945	2230 Main Street
South End Sewage Treatment Plant	49.79485543	−97.09636602	100 Ed Spencer Drive
West End Sewage Treatment Plant	49.84759578	−97.32364236	7740 Wilkes Ave

## DISCUSSION

### Taxonomic composition of DNA and RNA viruses

Since the biology of DNA phages has been more studied (16, 17), many phages identified to date are tailed bacteriophages grouped under the order *Caudovirales*. In the same context, *Caudovirales* represented the largest order of bacteriophages and thus viruses reported here. Other non-enveloped viruses such as *Autographiviridae*, *Kyanoviridae*, and *Peduoviridae* were present to a lesser extent across seasons (Fig. 2). Since non-enveloped viruses possess a highly resistant protein capsid that increases their survival to extreme environmental conditions and treatments such as heat, moisture, pH, and chemical detergents (18), they can withstand seasonal variations. In this context, portal and T4 major head proteins were the most abundant among DNA viruses along the rivers. Portal proteins are essential for phage assembly, facilitate DNA packaging, host cell delivery, and genome release (19), while bacteriophage T4 major head proteins (Gol peptide) bind elongation factor Tu (EF-Tu), creating a substrate for Lit protease to halt translation and induce cell death (20). Additional analysis using PHASTER detected phages such as *Pseudomonas* phage UFV-P2, *Rhizobium* phage 16-3, *Synechococcus* phage S-CBS3, *Thalassomonas* phage BA3, Cyanophage MED4-117, and *Ralstonia* phage RSK1 DNA. However, due to the sensitivity of sequencing (depth) of the PHASTER database, these phage sequences were only detected Summer 2021 (Fig. S1). This may indicate that the composition of phages may be affected by changes in weather. Although no statistical analyses were performed in the present study to confirm seasonal variability of phages, many environmental studies have observed marked seasonal fluctuations of microbial communities in aquatic environments with low phage concentrations in the winter and high concentrations in the summer (21–23). During the winter, there are reduced bacterial metabolisms, degeneration of organic matter and mobilization, as well as a high proportion of damaged cells, and as a result, lysogeny becomes the preferred strategy for phages to maintain their density (23). In northern Canada, lakes begin to change temperature at the beginning of fall—November. It is possible that weather conditions and factors during the spring are more favorable for optimal survival for a diverse range of phages compared to Fall 2021. Whereas DNA phages have been widely studied and successfully isolated from different aquatic environments, very few studies have successfully isolated RNA phages from varying aquatic environments, such that only two families, *Cystoviridae* and *Leviviridae,* of RNA phages have been discovered to date (24). In 2016, a study by Krishnamurthy et al. (25) identified 122 partial genomes of RNA *Cystoviridae* phages from a transcriptome of pure culture of *Streptomyces avermitilis*. This publication was one of the first studies to identify RNA phages based on their affinity for a gram-positive bacteria, which highlights the issue of many RNA phages that remain undiscovered in comparison to DNA phages (25). Although RNA phages were not classified using geNomad, BLAST, and PHASTER, protein-coding genes identified from assembled reads of RNA viruses revealed a consistent abundance of RNA phage-related functionalities such as long tail fiber protein (1%–5%), putative tail protein (2%–5%), and RIIA lysis inhibiter (1%–11%) present during the Fall of 2021 (Fig. 6). This indicates that the unclassified RNA viruses may contain phages belonging to *Cystoviridae* and *Leviviridae* families.

### Evaluating seasonal variabilities of DNA and RNA viral communities

An exploratory factor analysis revealed that RNA viruses such as *Picobirnaviridae, Tombusviridae,* RoV*,* and *Astroviridae* were observed within the same quadrants during sampling events for sites 6–8 collected along the Assiniboine River (Fig. 7). On the contrary, DNA viruses such as *Mimiviridae, Drexlerviridae,* crAssphage, HAdV, *Straboviridae, Peduoviridae*, and bacterial *16S rRNA* were found within the same quadrant during spring for locations 1–5 and 9–11 along the Red River (Fig. 7). Sampling was conducted at locations 1–5 and 9–11 along the Red River from areas of lesser polluted environments to those that were more influenced by anthropogenic activities including wastewater discharges. Sampling of this nature was used to assess the influence of urbanization in these areas. We hypothesize that the abundance of DNA and RNA viruses was influenced by Factor 1 or “land use,” where the viral DNA community in the Red River was influenced by urbanization and industrialization in Winnipeg. On the other hand, RNA viromes in the Assiniboine River were shaped by agricultural activities from upstream communities such as Portage la Prairie, Brandon, South Headingly, and small rural municipalities.

We propose that the distinct clusters of sampling sites 6–8 in the Assiniboine River observed in all seasons (Fig. 7) contained viromes that were largely influenced by agricultural practices from communities situated upstream to the Assiniboine River. The Assiniboine River (1,070 km long) begins in eastern Saskatchewan and flows eastward through Brandon, Portage la Prairie, and Headingley. Industrial wastewater generated from major manufacturing plants in these areas may contain industrial pollutants such as TSS, oil, grease, alkaline pH, bacteria, selenium, chemical compounds, and heavy metals (26). Although treatment processes at the aforementioned municipalities help to reduce the contaminants in industrial wastewater, we observed correlations in our exploratory analysis between water quality parameters pH, TSS, and DO and sampling locations 6 and 7 along the Assiniboine River during the Summer 2021 (Fig. 7). These results confirm the notion that these parameters are influenced by industrial and agricultural discharges. TSS was found to be positively correlated with HAdV (*r* = 0.41634, *P* = 1.60E – 02) and *Birnaviridae* (*r* = 0.35087, *P* = 4.53E – 0) (Fig. 7 and 8). High concentrations of TSS (above 40 mg/L) promote the survival of waterborne microorganisms such as bacteria and viruses (27), which can withstand chemical disinfectants by protecting themselves in TSS aggregates.

Factor 2 or “day length and temperature” was the most influential factor on the majority of the DNA and RNA viral communities identified in the study. Day length and temperature were positively correlated with each other (*r* = 0.52809, *P* = 1.60E – 03), and as a result, both factors exhibited a similar influence on the viral community (Fig. 7 and 8). High temperatures increase the viral inactivation rate due to the denaturing of the secondary structures of proteins leading to impaired molecular viral function (27, 28). In the present study, average temperatures of water collected across each season were lowest (0°C) in the fall and highest (18°C) in the summer (results not shown), none of which were extreme temperatures that altered the stability of viromes as high temperatures of 56°C are required for viral inactivation due to the stability of enteric viruses (29, 30).

Day length is another environmental factor known to increase viral inactivation. When UV light penetrates the cell walls of microorganisms, the molecular structure becomes permanently altered, rendering them inactive and non-infectious. Treatments designed to screen and remove fecal coliform, *E. coli,* and waterborne parasites *Cryptosporidium* and *Giardia* from wastewater at Winnipeg’s WWTPs often include chlorine coupled with UV light disinfection due to lower associated commercial treatment costs. In the present study, the average day length during the spring was 15 hours, while that of summer and fall was 13 hours and 8 hours, respectively (Table S3). Since there was a gradual decrease in day length across seasons, we propose that viral inactivation was highest during the Spring and lowest during the Fall of 2021. This was further confirmed in Fig. 7, which evidenced a close relationship was observed between day length, temperature, and the spring and summer sampling events.

In the present study, the flow rate of river water was found to be weak but positively correlated to *Chaseviridae* (*r* = 0.38404, *P* = 2.74E − 02), *Demerecviridae* (*r* = 0.30271, *P* = 8.68E − 02), *Drexlerviridae* (*r* = 0.37265, *P* = 3.27E − 02), and *Mimiviridae* (*r* = 0.35661, *P* = 4.16E − 02) (Fig. 7 and 8). The effluent discharge was fastest (~2.04 m/s3 and 1.82 m/s3) during Spring 2021 sampling event at sites 1–5 and 9–11 along the Red River. This flow may have disturbed particulate matter such as clay, dirt, and soil from sediments settled along the riverbed and resuspended these within the waterbody as the river flows along its course. The Assiniboine River merges into the Red River at a central point along the Red River, which is represented in our study as location 5 (Fig. 12). Thus, location 5 is subjected to an increase in flow rate as well as downstream locations 9–11 which receive a combined flow from the Assiniboine River and the southern end of the Red River (Fig. 12). The taxonomy of viruses at these locations indicated that during the Summer 2021, locations 5 and 9–11 consisted of approximately 1%–2% relative abundance of *Chaseviridae*, *Demerecviridae*, *Drexlerviridae,* and *Mimiviridae* (Fig. 2), thereby confirming their positive correlation to effluent discharge. Our taxonomic classifications also indicated that these viruses were least present during the Spring and Fall 2021 sampling events (Fig. 1 and 2). *Chaseviridae*, *Demerecviridae*, *Drexlerviridae,* and *Mimiviridae* are non-enveloped or “naked viruses,” which are highly resistant to wastewater treatment processes and environmental stressors due to their protein capsid that remains intact (18). Therefore, these viruses may be stable in high flows of water and can be transported to downstream locations (31). Continuous sampling events and sequencing are required to make an accurate inference on the effect of water flow rate on the viral community of the Red River. Although water flow rate was not found to significantly influence the transport of most viromes identified, environmental studies have highlighted its importance (31, 32).

Longitudinal analysis of viral (HAdV, PMMoV, crAssphage, and RoV) and bacterial (*uidA* and *16S rRNA* genes) markers revealed striking differences between sample location sites. GCNs of HAdV, crAssphage, and bacterial *16S rRNA* in orders of magnitude of 10^2^–10^3^ (volume) were detected in sample sites along the Red and Assiniboine rivers and exhibited significant changes across seasons—Spring, Summer, and Fall of 2021 (*P*-values of 1.00E − 04 and 4.85E − 02) (Fig. 9). Although HAdV is prevalent all year-round, HAdV outbreaks are commonly reported in late winter and early spring (33). Gene copies per volume (100 mL) quantified for HAdV were highest during the Spring 2021 sample collection event and lowest during the Fall (Fig. S8), which thereby aligns with current literature and explains the seasonality of the virus identified in Fig. 9.

Interestingly, crAssphage was found to be seasonally variable in the present study (*P*-value = 4.85E − 02). However, environmental studies suggest that the bacteriophage does not have a pronounced seasonal pattern and is equally disseminated in raw sewage and treated wastewater (34–36). crAssphage constitutes the majority of the viral fraction of the human gut microbiota colonizing infants from as early as 1–3 months, after which the phage becomes more widespread throughout the guts of toddlers between the ages of 2 and 5 (37). The stool of 1-month-old infants contains, on average, 1.6 × 10^9^ virus-like particles per gram of stool, which increases to 9.4 × 10^8^ per gram of stool in toddlers aged 2–5 and in adults (37). Since crAssphage is naturally occurring in the intestines of adults and infants, its frequency in human feces and thus wastewater occurs at high concentrations without seasonal fluctuations. Although a significance was observed at the 95% confidence level, a *P*-value of 4.85E − 02 indicates that the seasonality of crAssphage in our study may be due to slight variabilities in the viral concentration at the locations in which the samples were collected (Fig. 9).

Quantitative analysis revealed consistently highest concentrations of PMMoV in orders of 10^3^ copies/100 mL at more than 60% of sample collection locations during the sampling events (Fig. S10). PMMoV has been detected at higher concentrations in municipal wastewater from all of the world more than any other human enteric virus (38, 39), and in the present study, we have also shown that the quantities of PMMoV (10^4^ copies/100 mL) detected along the Red and Assiniboine rivers were higher than that of HAdV and crAssphage, which were in orders of 10^2^ copies/100 mL (Fig. S8, S9, and S10). The abundance and persistence of PMMoV in aquatic environments is largely attributed to the fact that its presence in human feces is of dietary origin and is therefore excreted from a large population of healthy individuals. PMMoV frequent entry into municipal wastewater and reclaimed water occurs all year-round without any substantial seasonal fluctuations (34, 40). The time series analysis from our study aligns with current literature that PMMoV is not seasonally variable (*P*-value = 13.68E − 01) (Fig. 9). The number of RoV cases of infection has since declined due to the widespread administration of the RotaTeq and Rotarix vaccines within Canada. Since RoV outbreaks are becoming more sporadic within recent years, we suggest that this may be the reason the virus showed no seasonal pattern (*P*-value = 6.96E − 01) (Fig. 9; Fig. S11).

To further examine the seasonal differences of sequenced samples, the beta diversity of populations of DNA and RNA viruses between sample locations as well as the alpha diversities of viral communities within each sample collection site were measured. To avoid batch effects and confounding events, samples were processed and sequenced at the time of collection during the Spring, Summer, and Fall 2021 sampling events. Therefore, we are certain that our data are of high quality, and that our analysis indicates true seasonality. From our analysis, the DNA viral communities in the Red River at locations 1–5 and 9–11 identified during the Spring of 2021 were found to be most similar (Bray-Curtis dissimilarity distance of 5.73E − 02) to those identified during the Fall of 2021 (Fig. 10). Although Spring and Fall are seasons with contrasting characteristics, both consist of moderate temperatures ranging from −5°C to 20°C and high precipitation, which may shape the microbial community in aquatic environments. Approximately 59.2% (16/27) of viruses assessed in the present study observed a negative correlation with rain, while a weak but positive correlation was observed for the remaining viruses (Fig. 8). Studies suggest that prolonged periods of rainfall may dilute pathogen concentrations in surface water (41) as well as alter the viral concentrations from surface water runoff and resuspension of bottom sediments (42, 43).

As previously outlined, the effect of urbanization in Winnipeg may have shaped the viral community in the Red River, while agricultural practices from communities such as Portage la Prairie, Brandon, and small rural communities influence the viral community in the Assiniboine River. Given that the summer months are marked by warmer temperatures and increased sunlight, we would expect a reduction in the microbial community as these factors increase viral inactivation (27). However, the stability of DNA viruses may also increase resistance to environmental stressors.

On the contrary to the seasonality of DNA viruses, the RNA viral communities present in the Red and Assiniboine rivers during the Spring and Fall were evidenced to be more closely related (low Bray-Curtis dissimilarity distance of 5.73E − 02) compared to those present in the Summer (Fig. 10). Although Spring and Fall appear to be characteristically contrasting seasons, the two maintain some similarities. Spring and Fall relatively maintain cooler temperatures compared to that in the Summer. In our present study, temperatures during the spring sample collection ranged from 15.2°C to 16.1°C, while those during the fall ranged between 0.9°C and 13.4°C (Table S3). In addition, both spring and fall maintain roughly equal daylight and nighttime hours due to the earth’s equinoxes (44). In Winnipeg, the average day length during Spring 2021 was 15 hours, while that of Summer and Fall of 2021 was 13 hours and 8 hours, respectively (Table S3). These reasons, therefore, allow for minimal seasonal change in the microbial diversity.

To assess the microbiome diversity within a given sequenced sample, the alpha diversity indices, Simpson’s (1-D) and Shannon (H), were measured for DNA and RNA viral communities at each sample location. In comparison to RNA viral communities, which were found to be more uniform due to lower index values for Simpson’s (1-D) and Shannon (H) indices, DNA viruses measured high values for both indices, which indicated high species diversity among all surface water samples collected (Table 2). We acknowledge some limitations in the filtration and precipitation methods used in this study to concentrate viruses. For instance, DNA viruses such as adenovirus are close to 100 nm (45), and tailed bacteriophages within the class Caudoviricetes such as T4-like Myoviridae and Enterobacteria phage P1 can be over 100 nm (46, 47). Enveloped viruses such as Severe acute respiratory syndrome coronavirus 2 (SARS-CoV-2), some influenza viruses, and other coronaviruses are also larger than 100 nm in size (48). In addition, viruses such as *Orthomyxoviridae*, *Picornaviridae*, *Reoviridae*, *Fiersviridae*, and *Adenoviridae,* to name a few, tend to form aggregates in infected cells (49) or synthetic membranes by electrostatic and hydrophobic forces (50). These aggregates (>300 nm) and other giant viruses may have been adsorbed in the 0.1 µm and 0.2 µm membrane disk filters. Although there is no consensus when it comes to viral concentration methods from complex environmental samples (51), the utility of skimmed milk flocculation (SMF) has been demonstrated to concentrate DNA and, particularly, RNA viruses (52, 53). Within this context, many RNA viruses are still yet to be discovered, DNA viruses have been more extensively studied and are found to contain considerable diversity especially among their genome structure. While RNA viruses maintain the advantage of high genetic diversity due to their increased susceptibility to mutation rates, DNA viruses maintain a higher species diversity than RNA viromes. This is because DNA viruses are classified with large genome sizes ranging from ≤2 kb up to 375 kb DNA (54). Furthermore, dsDNA viruses have been shown to contain more size-flexible genome distribution than ssDNA viruses (55). Regardless, the diverse range of genome size distribution increases the diversity of organisms which DNA viruses can infect. Our findings, which display a higher species diversity for DNA viruses than RNA viruses, thereby confirm current literature. Our results from Tukey-Kramer comparisons across seasons indicated that there are marked seasonal patterns in the species richness and evenness of DNA viral communities (*P*-values of 1.00E − 04). In the present study, we observed the highest species diversity and richness of DNA viral communities during the Fall and Summer of 2021 (Fig. 12; Fig. S4). Viruses typically display marked seasonal peaks, although they may be present throughout the year. It is possible that the conditions of fall and summer may be favorable to host diverse DNA viral communities in the Red and Assiniboine River. DNA viruses were found to be more abundant than that of RNA viruses among aquatic samples collected in our study, possibly because DNA viruses maintain a high genomic stability due to their ability to use the host cell 3′-exonuclease proofreading activity to repair common kinds of DNA damage that will lower their mutation rates (56, 57). Additionally, many of the highly abundant DNA viruses identified in our study are non-enveloped in structure and possess a very resistant protein capsid known to increase their survival to extreme environmental conditions and treatments such as heat, moisture, pH, and chemical detergents (18). RNA viruses may be less abundant and highly variable across seasons because their polymerases lack proofreading components necessary for error correction and repair, making them highly error-prone (56, 57). In conclusion, the relative abundances of DNA and RNA viruses identified were impacted by seasonality to a greater degree than the species diversities observed in each sample.

This study aims to address the demand for aquatic reform. Most infectious diseases of public health concern are caused by viruses. More than 100 different pathogenic viruses are excreted in human and animal wastes that are then disseminated further into the aquatic environment (14). Waterborne pathogens continue to emerge in effluents from urban or agricultural discharges or non-point sources, despite current testing at treatment plants. As of March 2021, there were 164,289 registered indigenous people in Manitoba belonging to First Nations, of which approximately 60% live on reserves (58). From a total of 63 First Nations Reserves located in Manitoba, 10 are suited along the shores of Lake Winnipeg where local natives depend on this flow of water for domestic and recreational needs such as hydroelectricity, fishing, industrial or agriculture usages, or any household needs. Since Manitoba waterways are interconnected—the Assiniboine River drains into Red River, which then drains into Lake Winnipeg—contamination of either river has an impact on Lake Winnipeg. Since viruses are more pathogenic than bacteria, knowledge of these in treated effluents of the Red and Assiniboine rivers is imperative to aquatic health and, subsequently, the health of many First Nation communities relying on surface waters for domestic and recreational usages.

Future directions of this research project require continuous sequencing and validation methods over longer sampling event periods to accurately establish the characteristics and dynamics of the viral community structures in aquatic environments influenced by anthropogenic activities. This may ultimately lead to the development of novel viral markers of fecal contamination that could be used complementary to current microbial indicators.

## MATERIALS AND METHODS

### Surface water sampling

To evaluate seasonal variabilities, aquatic sampling was conducted at 11 locations along the Red and Assiniboine rivers in the Spring on May 16th, Summer on August 27th, and Fall on 21 November 2021. Of the 11 locations, 9 of them were located in the proximity or downstream of the discharge points from Winnipeg’s three major sewage plants. Two sample collection sites were used to represent a case-control study from undisturbed or lesser-polluted environments. Location 1 was upstream of the NEWPCC outfall in the Red River situated outside the Winnipeg Metropolitan area (Duff Roblin Provincial Park), while location 6 was situated upstream of the WEWPCC outfall in the Assiniboine River (Fig. 8; Table 2). As the numbers increase from the control sites, they are located further away from each of the wastewater treatment plants. In addition, MilliQ water was used as a negative background control. A total of 36 10 L surface water samples were analyzed over the three sampling event periods. All samples collected were stored in a 4°C cold room until processing occurred within 24 hours following sample collection.

### Concentrating viruses from environmental samples

To minimize noise during metagenomic sequencing from larger fractions such as micro-eukaryotes and bacteria to the smaller ones such as viruses, raw water samples were first subjected to capsule and vacuum filtration using PALL Corporation GWV high-capacity groundwater sampling capsules of 0.45 µm size and PALL membrane disk filters of 0.2 µm and 0.1 µm sizes. Microbial fractions were processed from the sampling capsules of 0.45 µm size as previously described (59). On the other hand, the filtrate underwent SMF, where the skimmed-milk particulates target the non-settling particles such as viruses and force them to clump together into larger and heavier solids known as “flocs” by means of sedimentation. A modified SMF approach described by Calgua et al. (60) and Fernandez-Cassi et al. (52) was used to concentrate virus particles (60, 61). A 1% weight-by-volume pre-flocculated skimmed milk solution was prepared using 1.32 L of RICCA synthetic seawater (ASTM D 1141 substitute ocean water without heavy metals) and 13.2 g of Difco skimmed milk powder and then acidified to a pH of 3.5 using 2 M HCl. A total of 100 mL of the pre-flocculated acidified skimmed milk solution was transferred to 10 L acidified (pH 3.5) environmental water samples (final skimmed milk concentration 0.01% [wt/vol]). Using a magnetic stirrer and a magnetic stir bar, samples were stirred for 8 hours at room temperature and allowed to settle down by gravity for an additional 8 hours. Without disturbing the flocs, the supernatant was carefully removed using a vacuum pump. The remaining flocs were aliquoted and balanced according to weight in 50 mL centrifuge tubes for centrifugation at 8,000 *g* for 30 min at 4°C. The resulting pellet, which theoretically contained viruses of interest, was dissolved in 0.2 M phosphate buffer. To eliminate free DNA and RNA present that may be co-precipitated into the kit used for nucleic acid extraction, the dissolved pellet was treated with DNase I and RNase A (Thermo Fisher Scientific, Waltham, MA, USA). As outlined by the manufacturer (Thermo Fisher Scientific), 3 µL of 10 mg/µL RNase A was added to the dissolved pellet and incubated at 37°C for 30 min. For DNase treatment, 30 µL of 10× reaction buffer with MgCl_2_ and 3 µL of 1 unit/µL DNase I were added to the pellet and incubated at 37°C for 10 min. To inactivate the DNase enzyme, 3 µL of 50 mM EDTA was added and incubated at 65°C for 10 min. Inactivation of the RNase A enzyme occurred through nucleic acid extractions.

### Extracting and quantifying total nucleic acids

A Qiagen All-Prep DNA/RNA power microbiome kit was used to extract total nucleic acids from the dissolved pellet. As outlined by the manufacturer, 600 µL of warmed solution of PM1, 450 µL effluent sample, 100 µL ultrapure phenol:chloroform:isoamyl alcohol (Invitrogen, Thermo Fisher Scientific), and 6 µL 2-mercaptoethanol (Fisher Chemical, Fisher Scientific) were added to a Qiagen powerbead tube. Using a vortex adapter, the power bead tubes were vortexed horizontally at maximum speed for 10 min and then centrifuged at 13,000 *g* for 1 min at 22°C. The supernatant was transferred to a clean 2 mL Collection Tube where 150 µL of IRS Solution was added and incubated at 4°C for 5 min. The 2 mL collection tubes were centrifuged at 13,000 *g* for 1 min. The flow-through was discarded, and ~700 µL of the supernatant was transferred to clean 2.2 mL collection tubes. To each collection tube, 600 µL solutions PM3 and PM4 were added and vortexed briefly. For each effluent sample, 625 µL of the supernatant was loaded into a membrane binding (MB) spin column and centrifuged at 13,000 *g* for 1 min. The flow-through was discarded, and the process was repeated until all the supernatant was loaded into the MB spin columns for each sample. To wash the membrane of the MB spin column, which in theory contains the extracted nucleic acids, 600 µL of solution PM4 was added and centrifuged at 13,000 *g* for 1 min. The flow-through was discarded, and the process was repeated with 600 µL of solution PM5 that was added to the membrane. To dry the membrane of the spin column, the empty column was centrifuged at 13,000 *g* for 2 additional minutes. To elute the total nucleic acids from the membrane of the spin column, 100 µL of warmed RNAse-free water was added to the center of the white column membrane and incubated for 2 min. The MB spin column was then centrifuged at 13,000 *g* for 1 min. The final volume of extracted nucleic acids (100 µL) was transferred to a clean collection tube and used for downstream applications. A Qubit 4 fluorometer (Invitrogen, Carlsbad, CA, USA) was used to quantify the total amount of extracted DNA and RNA from surface water samples (Table S1).

### Enriching viral DNA and RNA fractions

To enrich viral RNA and viral DNA fractions and avoid the degradation of RNA, which is less stable than DNA and prone to being degraded by exogenous ribonucleases, the final volume of the total extracted nucleic acids (100 µL) was separated into half. For each sample, 50 µL were treated with Turbo DNAse (Thermo Fisher Scientific, Waltham, MA, USA), and the other half (50 µL) was treated with Ambion RNase III (Thermo Fisher Scientific, Waltham, MA, USA). As outlined by the manufacturer’s protocol, 1 µL of 1 unit/µL RNase III and 5 µL of 10X RNase III reaction buffer was added to the viral DNA fraction and incubated at 37°C for 1 hour. To inactivate the RNase III, the DNA fraction was incubated at 95°C for 5 min. To reanneal the DNA, the sample tubes were placed on ice for 8 min. As indicated by the manufacturer’s protocol, 2 µL of 2 units/µL Turbo DNase and 5 µL of 10× Turbo DNase buffer was added to the viral RNA fraction and incubated at 37°C for 30 min. To inactivate the Turbo DNase, 5.5 µL of homogenized DNase inactivation reagent in a 1:10 proportion was added to the viral RNA fraction and allowed to centrifuge at 10,000 *g* for 1.5 min at 22°C.

The treated total RNA fraction contained viral RNA, messenger RNA (mRNA), small RNAs, and ribosomal RNA (rRNA). Therefore, a QIAseq FastSelect kit (Qiagen Sciences, Maryland, MD, USA) was used for rapid removal of bacterial and mammalian rRNA. As outlined by the QIAseq FastSelect 5S/16S/23S rRNA handbook from Qiagen, 1.5 µL of 12 µL FastSelect FH Buffer, 1 µL from 8 µL FastSelect 5S/16S/23S enzyme previously incubated at 37°C for 5 min, 1 µL of 12 µL FastSelect rRNA HMR enzyme, and 1.5 µL of nuclease-free water were all added to 11 µL of viral RNA fraction and incubated in a MiniAmp thermal cycler (Applied Biosystems, Waltham, MA, USA) for 5.5 min at 89°C, 2 min at 75°C, 2 min at 70°C, 2 min at 65°C, 2 min at 60°C, 2 min at 55°C, 2 min at 37°C, and 2 min at 25°C and then cooled down at 4°C. Homogenized QIAseq beads (19.5 µL) were added to the total volume vortexed and incubated for 5 min at room temperature. To separate the bacterial and mammalian rRNA from the QIAseq beads containing the RNA of interest, the tubes were placed on a magnetic rack for 2 min until the solution became clear and the beads were on the magnetic rack. The supernatant was carefully discarded without disturbing the QIAseq beads. A total of 1.5 µL of nuclease-free water and 19.5 µL of QIAseq bead binding buffer were added to the QIAseq Beads, vortexed, and incubated for 5 min at room temperature. The tubes were placed on a magnetic rack for 2 min until the solution cleared and the beads were on the magnetic rack. The supernatant was carefully discarded without disturbing the QIAseq beads containing the RNA of interest. To wash the QIAseq beads, 200 µL of 80% ethanol was added to the tubes, and the supernatant was discarded. The QIAseq beads were air-dried for 2 min until all residual supernatant had evaporated. To elute the viral RNA fraction of interest, 31 µL of nuclease-free water was added to the QIAseq beads. The supernatant was vortexed and incubated at room temperature for 2 min to appropriately hydrate the QIAseq beads. The tubes were placed on a magnetic rack for 2 min until the solution cleared and the beads were on the magnetic rack. The supernatant containing the RNA of interest had a final volume of ~29 µL. To obtain quantifiable amounts of viral RNA necessary for sequencing, whole transcriptome amplification was conducted using a REPLI-g cell WGA & WTA kit that enables amplification of viral DNA and RNA in parallel reactions. For DNA viral fractions, this step was not required, as quantifiable amounts were observed after the addition of Ambion RNase III enzyme to degrade ribosomal rRNA.

As outlined by the REPLI-g cell WGA & WTA handbook from Qiagen, 8 µL of lysis buffer was added to 13 µL of the final RNA fraction of interest and was incubated at 24°C for 5 min followed by 95°C for 3 min and cooled down at 4°C. To the lysed cell sample, 2 µL of gDNA wipeout buffer was added and incubated at 42°C for 10 min to ensure gDNA removal. Eight microliters of Quantiscript RT mix was freshly prepared using 4 µL RT/polymerase buffer, 2 µL H_2_O, 1 µL of 0.4 µg/µL oligo dT primer, and 1 µL Quantiscript RT Enzyme Primer for a single reaction. Eight microliters of Quantiscript RT Mix was then added to 23 µL of the lysed and unsheared cell sample (as described above), briefly vortexed, and incubated at 42°C for 60 min to allow for reverse transcription. To stop the reverse transcription reaction, the sample was incubated at 95°C for 3 min and cooled down at 4°C. Ten microliters of freshly prepared ligation mix was prepared from 8 µL 10× ligase buffer and 2 µL ligase mix for a single reaction. A total of 10 µL of ligation mix was added to 31 µL of the Quantiscript RT reaction, vortexed briefly, and incubated at 24°C for 30 min to allow for ligation. To stop the ligation reaction, the sample was incubated at 95°C for 5 min and cooled down at 4°C. Thirty microliters of REPLI-g SensiPhi amplification mix was freshly prepared from 29 µL REPLI-g Single Cell Reaction Buffer and 1 µL REPLI-g SensiPhi DNA Polymerase for a single reaction. Thirty microliters of REPLI-g SensiPhi amplification mix was added to 41 µL of the ligation reaction, vortexed briefly, and incubated at 30°C for 2 hours to randomly amplify the cDNA. To inactivate the DNA polymerase, the reaction was incubated at 65°C for 5 min and cooled down at 4°C. The final volume of randomly amplified cDNA (71 µL) was used to establish a baseline of virome distribution through metagenomics and preliminary screening of enteric viruses.

### Characterizing virome distribution

Metagenomics was applied to explore and characterize the DNA and RNA virome distribution in urban-influenced environments. Enteric viral markers quantitatively assessed in each surface water sample were *Human Adenovirus 40/41* (HAdV), *Cross Assembly Phage* (crAssphage), *Pepper mild mottle virus* (PMMoV), and RoV, as previous literature has evidenced these viruses to be relatively more stable enteric viral markers of fecal contamination (11). Using the results generated from these approaches, seasonal patterns of viral communities were then determined using exploratory factor analysis, distance metrics on the beta diversity index, and generalized linear model (GLM) on the alpha diversity indices.

### Metagenomic analyses

A total of 36 10 L environmental samples collected over the three sampling event periods, once nucleic acid was extracted, were prepared for sequencing. Fifteen microliters of viral DNA and randomly amplified RNA fractions were sent to Dalhousie University’s Integrated Microbiome Resource (IMR) for Illumina sequencing (Dalhousie University, Halifax, NS, Canada) (62). A mock community of pooled DNA and RNA viruses was also included to account for metagenomic sequencing controls. DNA viruses included adenoviruses (such as adenovirus viral soup extract and adenovirus 1) and myoviruses such as Myophage g20(+) M2 and Myophage g20(+) M3. RNA viruses included enteroviruses (such as enterovirus and *Enterovirus Coxsackie B2*) and *Heterosigma akashiwo* RNA virus. These viruses adhered identically to the protocol used to concentrate human enteric viruses from environmental water samples and were then pooled in equimolar concentrations for metagenomic sequencing at IMR (Dalhousie University, Halifax, NS, Canada) (54). As outlined by IMR, samples sent for sequencing were tagmented, amplified through PCR techniques, barcoded for identification, purified using columns or beads, and normalized using Illumina beads. To generate paired-end Illumina sequenced reads, a pooled library was prepared using an Illumina Nextera Flex kit and ~1 ng of DNA samples (62). Massive parallel sequencing was conducted using NextSeq 550 System (Illumina Technology) and NextSeq 550 System High-Output Kit to produce ~4 M Pair Ended reads and 2.4 Gb/sample sequence (62).

Geneious Prime bioinformatic platform was used to pre-process high-throughput sequencing reads necessary to facilitate identification of virome distribution. Preprocessing of high-throughput reads adhered to the Geneious Prime alignment and assembly workflow, which provided steps to enhance accuracy and reduce computation time required to assemble the raw reads (63–65). The paired read data provided by IMR as separate forward and reverse compressed gzip sequences in fastq format was imported into Geneious Prime. Using the set paired reads operation from the BBMerge function accessible through the pre-processing sequence menu, the forward and reverse sequences were paired for each sample. The raw reads were not trimmed in Geneious Prime as the adapter trimming quality control step was completed by IMR using Nextera Flex DNA prep. Duplicate, contained, and overlapping sequences were then removed from the data set using the Dedupe function containing dedupe python library and machine learning algorithms, available from the remove duplicate reads option in the sequence menu (63, 64). Using the BBMerge function as part of the BBtools suite found in the merge paired reads option in the sequence menu, two overlapping paired-end reads were joined together by overlapping detection to form a single read (63, 64). To down-sample reads in high-depth areas of a genome and eliminate false-positive variant calls, the reads were normalized and corrected for error using the k-mer-based bioinformatic function BBNorm, a member of the BBTools package (63, 64, 66). The pre-processed paired-end reads were assembled, and contigs were generated using the *de novo* assembly method within Geneious Prime. Gene prediction of assembled reads as DNA and RNA viruses was identified using Prodigal (67). Contigs larger than 500 bp were processed with geNomad v.1.8.0 (virus_score ≥ 0.7) (68) to identify viral contigs. The completeness and quality of the identified viral contigs were further assessed with CheckV v.1.0.3 (69), and the contigs that had no viral genes were discarded. The remaining contigs were accepted as viral contigs, and they were taxonomically classified using BLASTp v.2.15.0 (70) against NCBI Viral RefSeq database (release date: 9 May 2024) with the search criteria of pident ≥70 and e-value ≤1E − 5. For the taxonomic assessment of viral contigs, the outputs from geNomad v.1.8.0 and BLASTp were combined. The protein-coding genes in the identified viral contigs were first predicted with Prodigal v.2.6.3 in -meta mode (67) and then annotated with Metacerberus v.1.3.0 (71) using VOG and PROG databases. The BLASTp v.2.15.0 against NCBI Viral RefSeq database reports generated for each sample were compiled together and visualized in Tableau Desktop (Tableau version 2024.2.2) to depict the relative abundance (%) across each sample location and thus seasonal variabilities. Additional analysis was conducted using PHASTER (PHAge Search Tool Enhanced Release) (72) to screen for prophage sequences in the samples. To obtain a detailed summary of prophages, the contigs in a fasta format were uploaded into PHASTER and visualized using Tableau Desktop (Tableau version 2024.2) to investigate the seasonal variability of phages in Spring, Summer, and Fall of 2021.

### Quantitative analyses

Enzymatic treatments of nucleic acid extracts enriched for viral DNA were quantitatively assessed through real-time quantitative polymerase chain reaction (qPCR) to identify the DNA viruses—HAdV and crAssphage. As described by Garcia et al. (11), a 10 µL qPCR reaction contained for each DNA enteric virus contained 5 µL of Taqman environmental master mix 2.0, 500 nM of each specific primers, AdV-F, AdV-R, 056F1, and 056R1, and 250 nM of probes Adv-P and 056P1 (73, 74, Table S2). HAdV and crAssphage were subjected to the following targeted qPCR conditions: 50°C for 2 min and 95°C for 10 min, followed by 40 cycles of 95°C for 15 s and 60°C for 1 min. Nucleic acid extracts enriched for viral RNA with no random amplification (Table S1) step were assessed through reverse transcription quantitative real-time PCR (RT-qPCR) to quantify PMMoV and RoV. A 10 µL RT-qPCR reaction to identify the RNA enteric viruses of interest contained 2.5 µL of fast virus 1-step master mix (4×) (Life Technologies, Carlsbad, CA, USA), 500 nM of each specific primers, PMMV-FP1-rev, PMMV-RP1, NSP3-F, and NSP3-R, and 250 nM of each probe: PMMV-P and NSP3-P (75, 76, Table S2). PMMoV and RoV were subjected to the following RT-qPCR conditions: 50°C for 5 min and 95°C for 20 s followed by 40 cycles of 95°C for 3 s and 60°C for 30 s. In addition to the viral targets, *16S rRNA* and *uidA* genes were also estimated to assess bacterial and *E. coli* GCNs from the 0.45 µm capsules as previously described (11, 77, 78). To generate standard curves necessary for quantifying DNA and RNA viruses, gBlocks gene fragments were used for these assays. On the other hand, standard curves for quantification of *16S rRNA* and *uidA* genes were conducted using genomic DNA extracted from *Pseudomonas aeruginosa* (ATCC 10145) and *Escherichia coli* (ATCC 25922), respectively. Seven positive controls/standards were made from serial dilutions of gBlocks at 7.06 × 10^7^ copies/µL. A non-template control consisting of nuclease-free water (Promega Corporation, Fitchburg, WI, USA) was used for each assay.

A QuantStudio 5 real-time PCR system (Applied Biosystems, Waltham, MA, USA) was used to conduct quantification of enteric viruses and microbial targets from the environmental samples collected over the three sampling event periods. All qPCR and RT-qPCR reactions used 2 µL of template. For each sample screened for an enteric virus of interest, all positive controls and non-template controls were run in triplicate. For a sample to be considered positive, two out of three of its replicates had to be amplifiable. According to Public Health Ontario, the cut-off (highest Ct value) for positivity of a sample is at 38 cycles (79). Thus, those above 38 cycles (from a total of 40 cycles) were considered as a negative result. Raw output data were analyzed following the methodology described by Ritalahti et al. (77). GCNs per volume were determined from the following equation:


$$\frac{\text{gene copies per reaction mix}\times \text{total volume of nucleic acid extracted (100 µL)}}{\left(\text{volume of nucleic acid added to each qPCR well - plate}\times \text{volume of environmental sample (mL)}\right)}$$

### Statistical analyses to determine seasonal patterns of viral communities

To explore the strength of the hypothesized relationships between viral families and latent environmental conditions at each sample site across season, an EFA was conducted in Statistical Analysis System (SAS, version 9.4 for Windows) and visualized using Tableau Desktop (Tableau version 2024.2). The following logarithmic physicochemical and biological parameters were included in the EFA model: (i) publicly available metadata from Winnipeg’s WWTPs such as TSS (mg/L), BOD_5_ (mg/L), cBOD_5_ (mg/L), NH_4_-N (mg/L), Ortho-Phosphorous (mg/L-P), TP (mg/L), *E. coli* (MPN/mL), and TN (mg/L), (ii) environmental metadata obtained using a YSI probe such as temperature (°C), pressure (mmHg), salinity (psu), and dissolved oxygen levels (mg/L), and (iii) information from meteorological stations in Winnipeg, MB, located nearby each sampling site, such as cumulative precipitation (mm), daylight length (min), and discharge of water flow rate (primary sensor derived [m^3^/s]). The characteristics of effluent wastewater samples for the dates the samples were collected are summarized in Table S3. Orthomax rotation that best fits all variables assessed in this study was used on the EFA model. A scatter plot was generated to visualize the factor loadings for the environmental parameters in relation to each sample site and season. A correlogram was also generated to assess the strength of the relationship between the water quality parameters and the relative abundance of the top 10 viral DNA and RNA communities identified metagenomically alongside the GCNs of enteric viruses assessed quantitatively.

Alpha diversity indices of the identified viral contigs were calculated using MerCat2 (-k 10 -n 8 -c 10) (80). Beta diversity of the identified viral contigs was calculated using the k-mer tables generated by MerCat2 with factoextra and MASS packages in RStudio (81). Alpha and beta diversities were visualized in Tableau Desktop (Tableau version 2024.2) as tables and scatter plots, respectively, to depict seasonal variabilities. A PROC GLM test and log10 transformation were used to calculate the statistical differences of each alpha diversity index between sample locations and between seasons of each sample collection period. Tukey-Kramer comparisons among sites and seasons were used in the SAS (version 9.4 for Windows) (82). To determine the similarities and dissimilarities between samples collected during the Spring, Summer, and Fall 2021, the Bray-Curtis distance matrix from the *Abdiv* package in R and R Studio (81) was calculated.

## SUPPLEMENTAL MATERIAL

Supplemental material aem.00408-24-s0001.docx Tables S1 to S3; Fig. S1 to S11.

ASM does not own the copyrights to Supplemental Material that may be linked to, or accessed through, an article. The authors have granted ASM a non-exclusive, world-wide license to publish the Supplemental Material files. Please contact the corresponding author directly for reuse.
