# Supplementary material for: Seasonal patterns of viromes in urban aquatic environments of Manitoba
Source: Appl Environ Microbiol. 2025 Sep 8;91(10):e00408-24. doi: 10.1128/aem.00408-24 (PMC12542761; doi:10.1128/aem.00408-24)
Supplement: Supplemental material — Tables S1 to S3; Fig. S1 to S11. [file aem.00408-24-s0001.docx]

**Supplemental Materials**

**Table S1**: Qubit 4 Fluorometer quantification of total nucleic acids (DNA and RNA) in eleven 10L samples collected during the Spring, Summer and Fall of 2021 at different locations along the effluents of the Red and Assiniboine River within Winnipeg, MB**.**

| Season  2021 | Sample Collection Location | Viral DNA (ng/uL) | Viral RNA (ng/uL) | |
| --- | --- | --- | --- | --- |
|  |  |  | **Viral RNA** | **cDNA** |
| Spring | 1 (Control/ Undisturbed Environment) | 12.80 | <0.025 | 17.50 |
|  | 2 | 25.00 | <0.025 | 1160 |
|  | 3 | 31.00 | <0.025 | 80.00 |
|  | 4 | 6.700 | <0.025 | 143.00 |
|  | 5 | 12.60 | <0.025 | 61.80 |
|  | 6 | 18.80 | <0.025 | 25.00 |
|  | 7 | 17.10 | <0.025 | 112.0 |
|  | 8 | 58.60 | 4.00 | 47.80 |
|  | 9 | 9.660 | <0.025 | 125.00 |
|  | 10 | 13.60 | <0.025 | 84.600 |
|  | 11 | 6.580 | <0.025 | 75.600 |
|  | Background control (MilliQ Water) | 0.0208 | <0.025 | <0.025 |
| Summer | 1 (Control/ Undisturbed Environment) | 22.40 | <0.025 | 314.00 |
|  | 2 | 40.40 | 4.000 | 318.00 |
|  | 3 | 35.80 | 4.200 | 302.00 |
|  | 4 | 39.60 | 4.000 | 256.00 |
|  | 5 | 35.60 | <0.025 | 258.00 |
|  | 6 | 26.20 | <0.025 | 78.00 |
|  | 7 | 7.180 | <0.025 | 141.00 |
|  | 8 | 23.00 | <0.025 | 43.00 |
|  | 9 | 31.00 | 4.400 | 314.00 |
|  | 10 | 62.600 | 4.000 | 300.00 |
|  | 11 | 102.00 | 7.340 | 320.00 |
|  | Background control (MilliQ Water) | 0.550 | <0.025 | <0.025 |
| Fall | 1 (Control/ Undisturbed Environment) | 21.800 | <0.025 | 242.00 |
|  | 2 | 32.800 | <0.025 | 348.00 |
|  | 3 | 23.000 | <0.025 | 412.00 |
|  | 4 | 23.200 | <0.025 | 326.00 |
|  | 5 | 20.400 | <0.025 | 402.00 |
|  | 6 | 68.200 | <0.025 | 354.00 |
|  | 7 | 30.000 | <0.025 | 294.00 |
|  | 8 | 34.400 | <0.025 | 398.00 |
|  | 9 | 23.200 | <0.025 | 386.00 |
|  | 10 | 25.400 | <0.025 | 81.800 |
|  | 11 | 35.200 | <0.025 | 378.00 |
|  | Background control (MilliQ Water) | 3.880 | <0.025 | <0.025 |

DNA viral quantities were quantified using a Qubit 1x double stranded DNA High Sensitivity Assay generating mass/concentration in each sample. RNA viral quantities were quantified using a Qubit RNA high sensitivity assay generating mass/concentration in each sample. As RNA quantities for most of these samples were below the detection limit of Qubit fluorometer (detection limit of 0.025ng/uL), RNA was then randomly amplified to generate cDNA which was quantified using a 1x double stranded DNA High Sensitivity Assay generating mass/concentration in each sample.

**Table S2:** Sequences of primers and probes used in targeted q-PCR and RT–qPCR assays to screen for DNA and RNA viruses of interest (modified from Garcia et al., 2022; (11)).

| **Assay** | **Primer/ Probe Name** | **Relevant Sequence (5’-3’)** | **Genomic Target** | **Source of Reference** |
| --- | --- | --- | --- | --- |
| Armored RNA | Arm_RNA-F | AGCCTGTCAATACCTGCACC | Coat protein from the RNA bacteriophage MS2 | Garcia et al., 2022 (11); |
|  | Arm_RNA-R | CACGCTTAGATCTCCGTGCT |  | Yanaç et al., 2022 (53) |
|  | Arm_RNA-P | Cy5-AGAGTATGAGAGGTCGACGA-TAO |  |  |
|  |  |  |  |  |
| 16S rRNA gene | Bac1055Y-F | ATGGYTGTCGTCAGCT | Bacterial isolates | Ritalahti et al., |
|  | Bac1392-R | ACGGGCGGTGTGTAC |  | 2006 (77) |
|  | Bac1115-P | FAM-CAACGAGCGCCNCAACCC-31ABkFQ |  |  |
|  |  |  |  |  |
| Rotavirus Type A | Tampere NSP3-F | ACCATCTWCACRTRACCCTCTATGAG | Non structure Protein 3 | Zheng et al., 2008 (76) |
|  | Tampere NSP3-R | GGTCACATAACGCCCCTATAGC |  |  |
|  | Tampere NSP3-P | VIC-AGTTAAAAGCTAACACTGTCAAA |  |  |
|  |  |  |  |  |
| Human Adenovirus 40/41 | AdV-F | GCCTGGGGAACAAGTTCAG | Hexon | Molecular Microbiology & |
|  | AdV-R | ACGGCCAGCGTAAAGCG |  | Genomics Team, British Columbia |
|  | AdV-P | NED-ACCCACGATGTAACCAC |  | Centre for Disease Control (2017) (73) |
|  |  |  |  |  |
| Cross Assembly phage (crAssphage) | 056F1 | CAGAAGTACAAACTCCTAAAAAACGTA GAG | Genomic base pair region: 14731 bp-14856 bp | Stachler et al., 2018 (74) |
|  | 056R1 | GATGACCAATAAACAAGCCATTAGC |  |  |
|  | 056P1 | FAM-AATAACGATTTACGTGATGTAAC |  |  |
|  |  |  |  |  |
| Pepper mild mottle virus (PMMV) | PMMV-FP1-rev | GAGTGGTTTGACCTTAACGTTTGA | 1878 bp-1901 bp^c^  and  1945 bp-1926 bp^c^ | Rosario et al., 2009 (75) |
|  | PMMV-RP1 | TTGTCGGTTGCAATGCAAGT |  |  |
|  | PMMV-P | FAM-CCTACCGAAGCAAATG |  |  |
|  |  |  |  |  |
| *Escherichia coli* | 784F | GTGTGATATCTACCCGCTTCGC | *uidA* | Frahm and Obst, 2003 (78) |
|  | 866R | AGAACGGTTTGTGGTTAATCAGGA |  |  |
|  | EC807 | FAM-TCGGCATCCGGTCAGTGGCAGT-BHQ1 |  |  |

**Table S3:** Treated wastewater quality parameters in the Red and Assiniboine Rivers observed during the Spring, Summer, and Fall 2021.

| **Season** | **Sample Location** | **DO (mg/L)** | **Precipitation (mm)** | **pH (units)** | **Daylength (mins)** | **TSS (mg/L)** | **BOD5 (mg/L)** | **cBOD5 (mg/L)** | **NH4-N (mg/L)** | **Ortho-Phosphorous (mg/L-P)** | **TP (mg/L)** | **TN (mg/L)** | **Salinity (psu)** | **Discharge (primary sensor derived) (m3/s)** | **E-coli (MPN/mL)** | **Water temperature (℃)** |
| --- | --- | --- | --- | --- | --- | --- | --- | --- | --- | --- | --- | --- | --- | --- | --- | --- |
| Spring | 1 | 10.07 | 0.12 | 6.59 | 928 | 11 | 22 | 10 | 1826 | 4.15 | 4.5 | 50.1 | 0 | 110 | 130 | 15.2 |
|  | 2 | 10.07 | 4.5 | 6.59 | 928 | 11 | 22 | 10 | 1826 | 4.15 | 4.5 | 50.1 | 0 | 110 | 130 | 15.2 |
|  | 3 | 10.07 | 4.5 | 6.59 | 928 | 11 | 22 | 10 | 1826 | 4.15 | 4.5 | 50.1 | 0 | 110 | 130 | 15.2 |
|  | 4 | 10.07 | 4.5 | 6.59 | 928 | 11 | 22 | 10 | 1826 | 4.15 | 4.5 | 50.1 | 0 | 110 | 130 | 15.2 |
|  | 5 | 10.07 | 4.5 | 6.59 | 928 | 11 | 22 | 10 | 1826 | 4.15 | 4.5 | 50.1 | 0 | 110 | 130 | 15.2 |
|  | 6 | 9.85 | 0.12 | 9.45 | 928 | 50 | 14 | 0.2 | 0.013 | 0.4 | 3.4 | 9.45 | 0 | 17.1 | 170 | 16.1 |
|  | 7 | 9.85 | 0.12 | 9.45 | 928 | 50 | 14 | 0.2 | 0.013 | 0.4 | 3.4 | 9.45 | 0 | 17.1 | 170 | 16.1 |
|  | 8 | 9.85 | 0.12 | 9.45 | 928 | 50 | 14 | 0.2 | 0.013 | 0.4 | 3.4 | 9.45 | 0 | 17.1 | 170 | 16.1 |
|  | 9 | 10.07 | 0.12 | 6.84 | 928 | 13 | 40 | 6 | 4892 | 3.06 | 4.3 | 46.9 | 0 | 66.4 | 40 | 15.6 |
|  | 10 | 10.07 | 0.12 | 6.84 | 928 | 13 | 40 | 6 | 4892 | 3.06 | 4.3 | 46.9 | 0 | 66.4 | 40 | 15.6 |
|  | 11 | 10.07 | 0.12 | 6.84 | 928 | 13 | 40 | 6 | 4892 | 3.06 | 4.3 | 46.9 | 0 | 66.4 | 40 | 15.6 |
| Summer | 1 | 10.72 | 15 | 6.74 | 825 | 8 | 21 | 4 | 2046 | 3.9 | 3.6 | 47.2 | 0 | 17.7 | 10 | 18.4 |
|  | 2 | 11.24 | 12.5 | 6.74 | 825 | 8 | 21 | 4 | 2046 | 3.9 | 3.6 | 47.2 | 0 | 17.7 | 10 | 14.4 |
|  | 3 | 9.88 | 12.5 | 6.74 | 825 | 8 | 21 | 4 | 2046 | 3.9 | 3.6 | 47.2 | 0 | 17.7 | 10 | 19.4 |
|  | 4 | 10.28 | 12.5 | 6.74 | 825 | 8 | 21 | 4 | 2046 | 3.9 | 3.6 | 47.2 | 0 | 17.7 | 10 | 19 |
|  | 5 | 8.07 | 12.5 | 6.74 | 825 | 8 | 21 | 4 | 2046 | 3.9 | 3.6 | 47.2 | 0 | 17.7 | 10 | 18.8 |
|  | 6 | 10.74 | 15 | 9.61 | 825 | 51 | 0 | 5 | 0.7 | 0.436 | 0.9 | 3.2 | 0 | 19.2 | 990 | 16 |
|  | 7 | 11.28 | 15 | 9.61 | 825 | 51 | 0 | 5 | 0.7 | 0.436 | 0.9 | 3.2 | 0 | 19.2 | 990 | 16.8 |
|  | 8 | 11.9 | 15 | 9.61 | 825 | 51 | 0 | 5 | 0.7 | 0.436 | 0.9 | 3.2 | 0 | 19.2 | 990 | 18.3 |
|  | 9 | 10.28 | 15 | 6.79 | 825 | 57 | 49 | 29 | 6837 | 3.35 | 4.7 | 41.2 | 0 | 37.1 | 110 | 18.3 |
|  | 10 | 10.27 | 15 | 6.79 | 825 | 57 | 49 | 29 | 6837 | 3.35 | 4.7 | 41.2 | 0 | 37.1 | 110 | 18.5 |
|  | 11 | 10.56 | 15 | 6.79 | 825 | 57 | 49 | 29 | 6837 | 3.35 | 4.7 | 41.2 | 0 | 37.1 | 110 | 17.6 |
| Fall | 1 | 1.1 | 0 | 6.85 | 526 | 9 | 14 | 7 | 1959 | 3.64 | 3.68 | 50.2 | 0 | 69.6 | 10 | 13.4 |
|  | 2 | 1.1 | 0 | 6.85 | 526 | 9 | 14 | 7 | 1959 | 3.64 | 3.68 | 50.2 | 0 | 69.6 | 10 | 13.4 |
|  | 3 | 14.88 | 0 | 6.85 | 526 | 9 | 14 | 7 | 1959 | 3.64 | 3.68 | 50.2 | 0 | 69.6 | 10 | 0.9 |
|  | 4 | 15.55 | 0 | 6.85 | 526 | 9 | 14 | 7 | 1959 | 3.64 | 3.68 | 50.2 | 0 | 69.6 | 10 | 0.1 |
|  | 5 | 15.03 | 0 | 6.85 | 526 | 9 | 14 | 7 | 1959 | 3.64 | 3.68 | 50.2 | 0 | 69.6 | 10 | 0.3 |
|  | 6 | 15.09 | 0 | 9.07 | 526 | 26 | 0 | 7 | 13.8 | 0.027 | 0.4 | 3.4 | 0 | 16.2 | 10 | 0.2 |
|  | 7 | 19.68 | 0 | 9.07 | 526 | 26 | 0 | 7 | 13.8 | 0.027 | 0.4 | 3.4 | 0 | 16.2 | 10 | 0.1 |
|  | 8 | 18.37 | 0 | 9.07 | 526 | 26 | 0 | 7 | 13.8 | 0.027 | 0.4 | 3.4 | 0 | 16.2 | 10 | 0.1 |
|  | 9 | 1.09 | 0 | 6.8 | 526 | 8 | 16 | 6 | 4301 | 3.73 | 4.7 | 43.2 | 0 | 93.7 | 20 | 13.4 |
|  | 10 | 14.87 | 0 | 6.8 | 526 | 8 | 16 | 6 | 4301 | 3.73 | 4.7 | 43.2 | 0 | 93.7 | 20 | 0.7 |
|  | 11 | 1.1 | 0 | 6.8 | 526 | 8 | 16 | 6 | 4301 | 3.73 | 4.7 | 43.2 | 0 | 93.7 | 20 | 13.4 |

BOD_5_ = five-day biochemical oxygen demand; DO= Dissolved Oxygen; TP= Total Phosphate.

DNA viral quantities were quantified using a Qubit 1x double stranded DNA High Sensitivity Assay generating mass/concentration in each sample. RNA viral quantities were quantified using a Qubit RNA high sensitivity assay generating mass/concentration in each sample.

**FIG S1:** Comparison of the 2021 DNA phage composition during the Spring, Summer and Fall of 2021 that were identified from PHASTER with complete scores and intact regions

**FIG S2:**  Factor analysis of viral DNA and RNA families and environmental variables observed at each sample collection site (1-11) during the Spring (blue dots), Summer (orange dots) and Fall (red dots) of 2021. Factor 1 represents the influence of agricultural activities from rural environments while factor 3 as seasonal influence of bacterial counts and viral markers on aquatic environments.


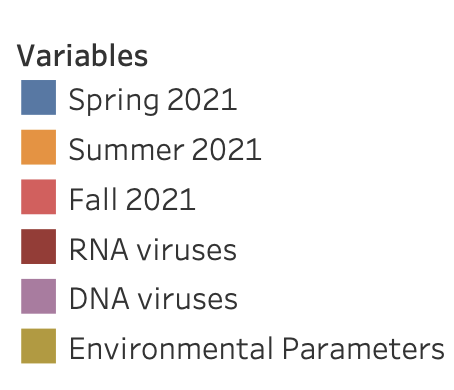

**FIG S3:**  Factor analysis of viral DNA and RNA families and environmental variables observed at each sample collection site (1-11) during the Spring (blue dots), Summer (orange dots) and Fall (red dots) of 2021. Factor 2 represents the effect of urbanization and industrialization practices on aquatic environments while factor 3 as seasonal influence of bacterial counts and viral markers on aquatic environments.

**FIG S4:** Assessment of Simpson (1-D) Alpha Diversity Index among RNA viral communities present in samples 1-11 collected along the Red and Assiniboine Rivers of Winnipeg during the Spring, Summer and Fall of 2021.

**FIG S5:** Assessment of Shannon Alpha Diversity Index among RNA viral communities present in samples 1-11 collected along the Red and Assiniboine Rivers of Winnipeg during the Spring, Summer and Fall of 2021.

**FIG S6:** Assessment of Simpson (1-D) Alpha Diversity Index among DNA viral communities present in samples 1-11 collected along the Red and Assiniboine Rivers of Winnipeg during the Spring, Summer and Fall of 2021.

**FIG S7:** Assessment of Shannon Alpha Diversity Index among DNA viral communities present in samples 1-11 collected along the Red and Assiniboine Rivers of Winnipeg during the Spring, Summer and Fall of 2021.

**FIG S8:** Abundance of *Human Adenovirus* per volume (100mL) (top panel) and per biomass (ng) (bottom panel) in aquatic samples 1–11 collected from the Red and Assiniboine rivers of Winnipeg, MB during (A) Spring, (B) Summer and (C) Fall 2021.


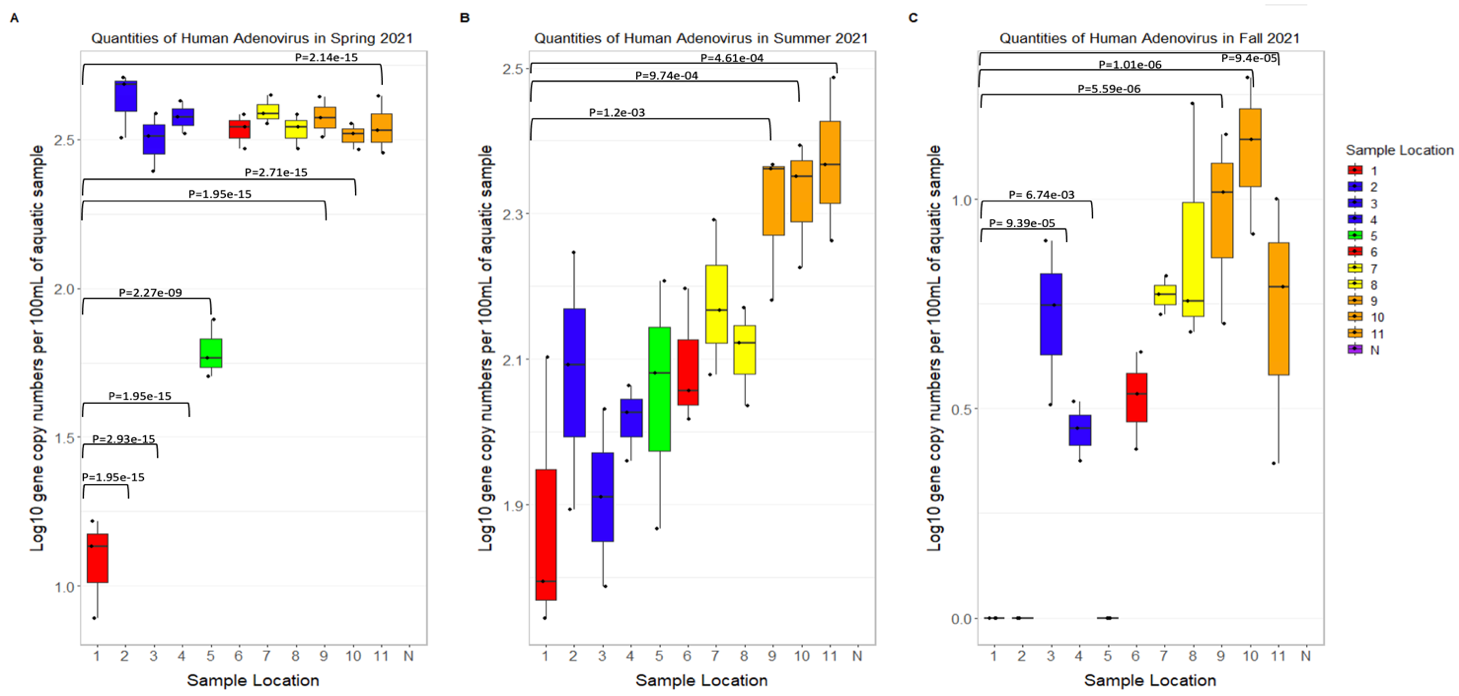

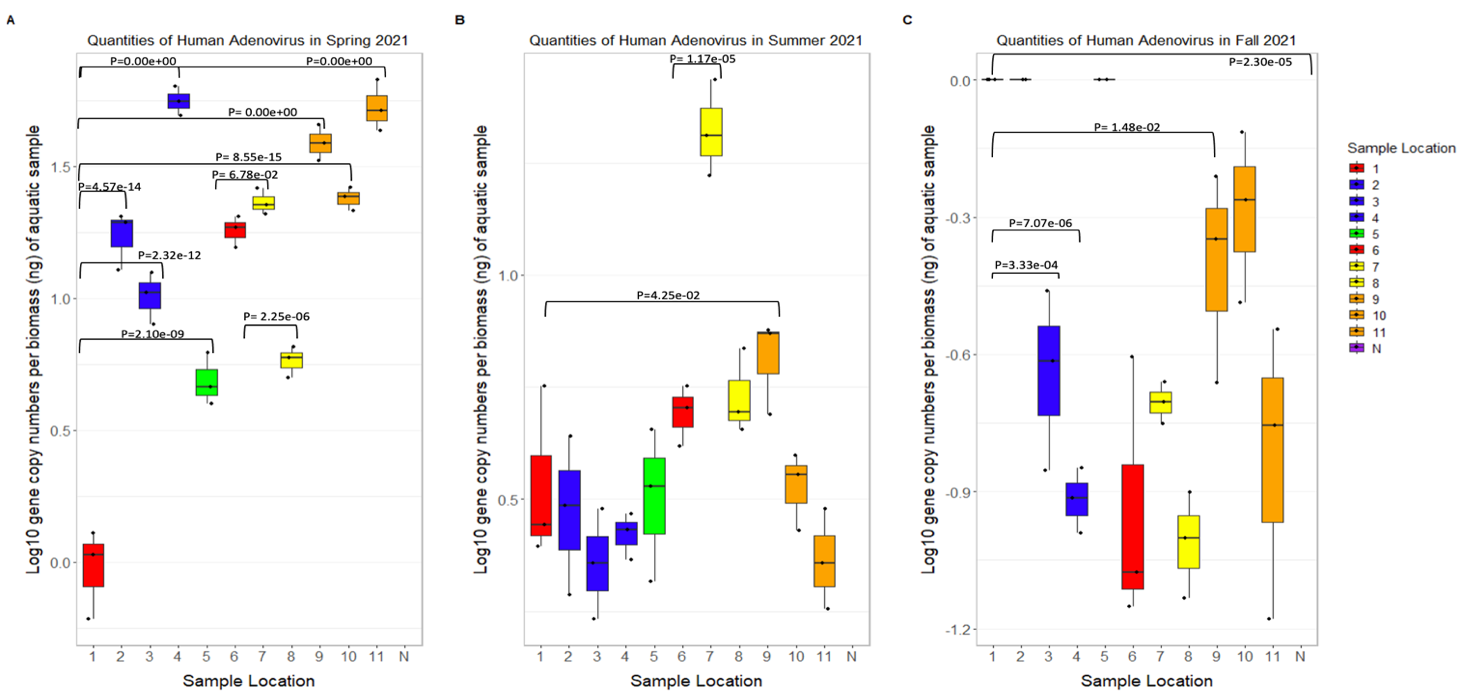


**FIG S9:** Abundance of *Cross-Assembly Phage* per volume (100mL) (top panel) and per biomass (ng) (bottom panel) in aquatic samples 1–11 collected from the Red and Assiniboine rivers of Winnipeg, MB during (A) Spring, (B) Summer and (C) Fall 2021.


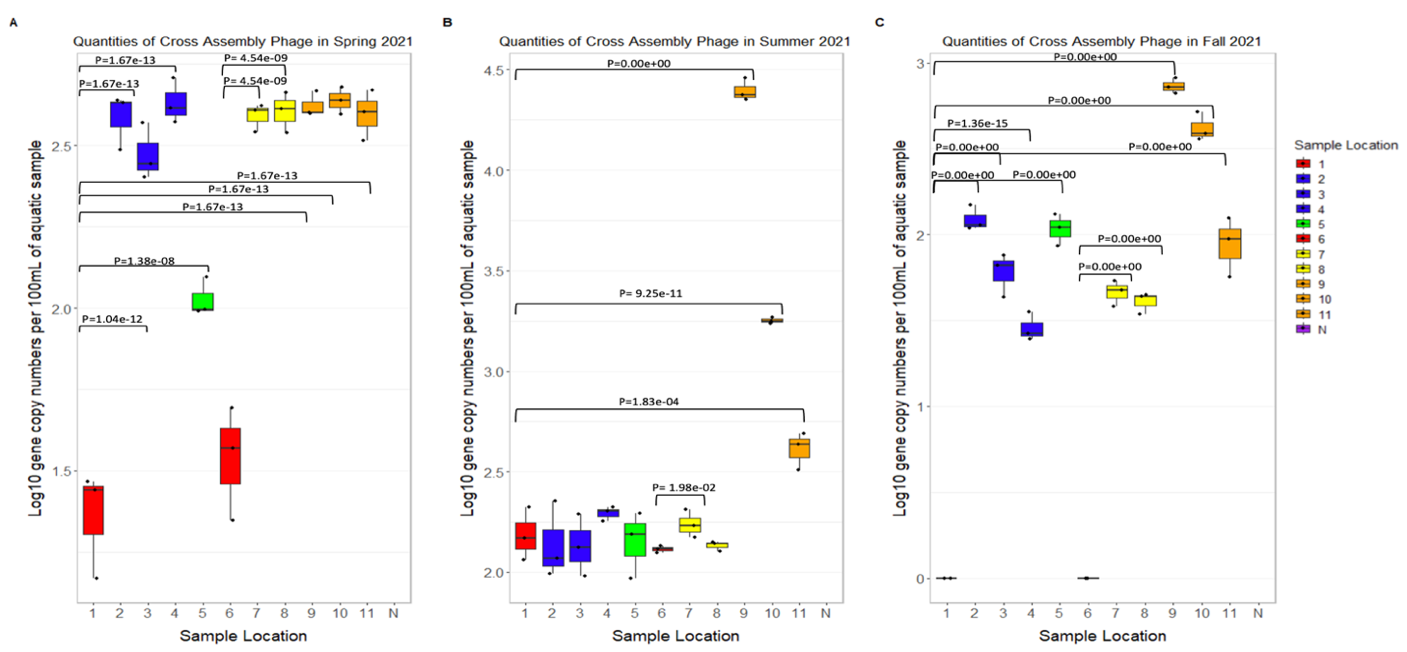

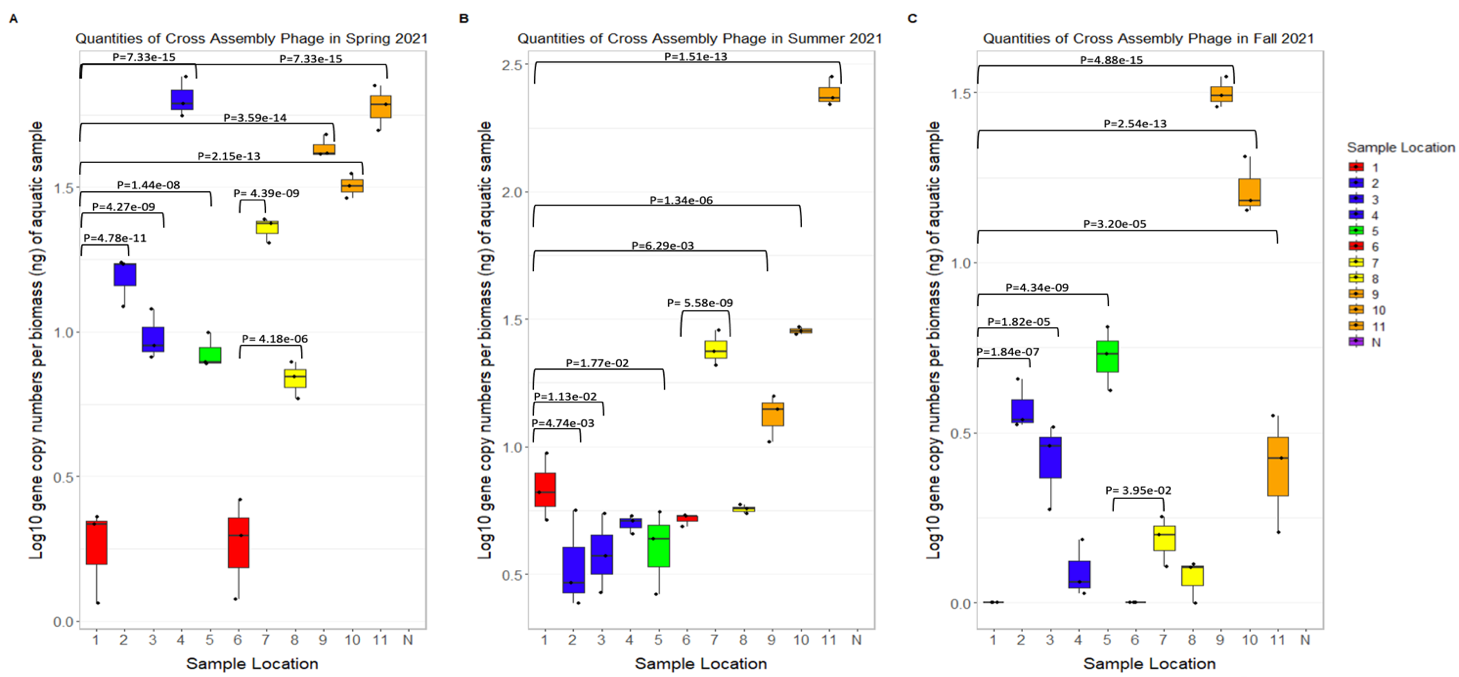


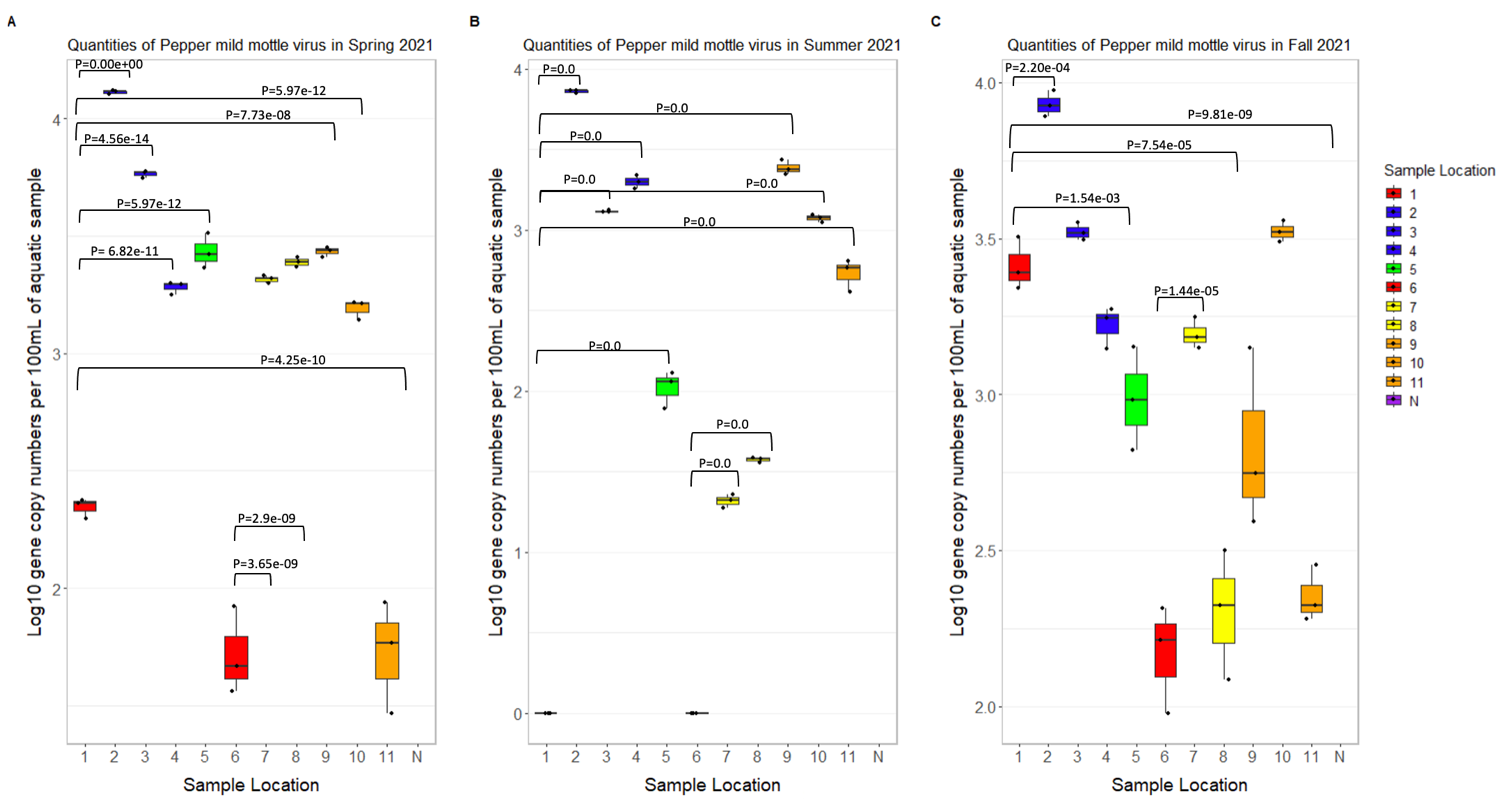
**FIG S10:** Abundance of *Pepper-mild mottle virus* per volume (100 mL) in aquatic samples 1–11 collected from the Red and Assiniboine rivers of Winnipeg, MB during (A) Spring, (B) Summer and (C) Fall 2021.


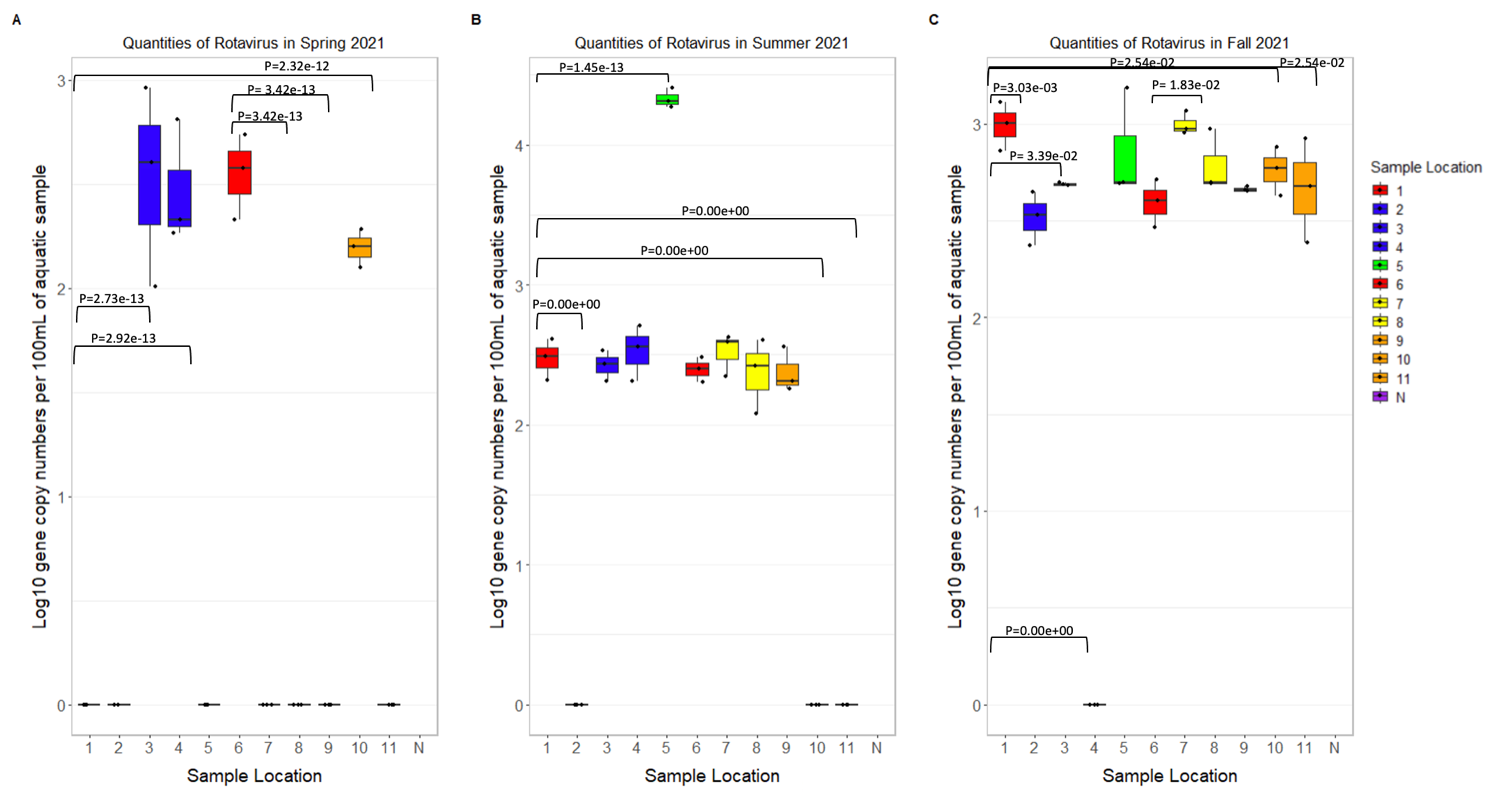
**FIG S11:** Abundance of *Rotavirus* per volume (100 mL) in aquatic samples 1–11 collected from the Red and Assiniboine rivers of Winnipeg, MB during (A) Spring, (B) Summer and (C) Fall 2021.
